# Facile Synthesis of Novel Coumarin Derivatives, Antimicrobial Analysis, Enzyme Assay, Docking Study, ADMET Prediction and Toxicity Study

**DOI:** 10.3390/molecules22071172

**Published:** 2017-07-13

**Authors:** Shailee V. Tiwari, Julio A. Seijas, Maria Pilar Vazquez-Tato, Aniket P. Sarkate, Kshipra S. Karnik, Anna Pratima G. Nikalje

**Affiliations:** 1 Y. B. Chavan College of Pharmacy, Dr. Rafiq Zakaria Campus, Rauza Bagh, Aurangabad 431001, India; shailee2010@gmail.com; 2 Departamento de Química Orgánica, Facultad de Ciencias, Universidad of Santiago De Compostela, Alfonso X el Sabio, Lugo 27002, Spain; julioa.seijas@usc.es (J.A.S.); pilar.vazquez.tato@usc.es (M.P.V.-T.); 3 Department of Chemical Technology, Dr. Babasaheb Ambedkar Marathwada University, Aurangabad 431004, India; aniketpharma1@gmail.com (A.P.S.); karnikkshipra@yahoo.co.in (K.S.K.)

**Keywords:** ionic liquid, antifungal activity, antibacterial activity, molecular docking, cytotoxicity, *in vivo* acute oral toxicity

## Abstract

The work reports the synthesis under solvent-free condition using the ionic liquid [Et_3_NH][HSO_4_] as a catalyst of fifteen novel 3-((dicyclohexylamino)(substituted phenyl/heteryl)-methyl)-4-hydroxy-2*H*-chromen-2-onederivatives **4a**–**o** as potential antimicrobial agents. The structures of the synthesized compounds were confirmed by IR, ^1^H-NMR, ^13^C-NMR, mass spectral studies and elemental analyses. All the synthesized compounds were evaluated for their *in vitro* antifungal and antibacterial activity. The compound **4k** bearing 4-hydroxy-3-ethoxy group on the phenyl ring was found to be the most active antifungal agent. The compound **4e** bearing a 2,4-difluoro group on the phenyl ring was found to be the most active antibacterial agent. The mode of action of the most promising antifungal compound **4k** was established by an ergosterol extraction and quantitation assay. From the assay it was found that **4k** acts by inhibition of ergosterol biosynthesis in *C. albicans*. Molecular docking studies revealed a highly spontaneous binding ability of the tested compounds to the active site of lanosterol 14α-demethylase, which suggests that the tested compounds inhibit the synthesis of this enzyme. The synthesized compounds were analyzed for *in silico* ADMET properties to establish oral drug like behavior and showed satisfactory results. To establish the antimicrobial selectivity and safety, the most active compounds **4e** and **4k** were further tested for cytotoxicity against human cancer cell line HeLa and were found to be non-cytotoxic in nature. An *in vivo* acute oral toxicity study was also performed for the most active compounds **4e** and **4k** and results indicated that the compounds are non-toxic.

## 1. Introduction

Many drug-resistant human pathogenic microbes have been observed in the past few decades [1] and this is becoming a serious public health problem in a wide range of infectious diseases [2,3]. In spite of a large number of antibiotics and chemotherapeutics available for medical use, antimicrobial resistance has created a substantial medical need for new classes of antimicrobial agents as these resistant pathogenic microbe strains cause antimicrobial treatment failure and enhance the mortality risks and sometimes contribute to complications. To overcome this problem the development of new bioactive compounds effective against resistant strains is highly needed and the design and synthesis of newer antimicrobials will always remain an area of immense significance [4,5]. One approach to the discovery of novel and potent antimicrobial agents is by modifying the structure of a well- known antimicrobial agent while the second strategy is to combine together two or more different antimicrobial pharmacophores into one molecule.

Coumarin is a class of heterocyclic compounds containing a benzene ring structure and an α-pyrone, a moiety commonly found in Nature and with multiple biological activities [6]. It is known that a wide range of medicinal plants (used since more than 1000 years ago) contain high levels of coumarins [7]. These compounds have become indispensable structural units that are useful in medicinal chemistry, displaying anticancer [8], antioxidant [9], anti-plasmodial [10], anti-malarial [11], anti-rhinovirus [12], antifungal [13] and antibacterial activity [14]. Much research has been focused on the inhibition of bacterial growth by naturally occurring coumarins such as xanthoxin, herniarin, umbelliferone and scopoletin. Umbelliferone, scopoletin, and coumarin also exhibit good antifungal activity [15].

The Mannich reaction is one of the most important carbon-carbon bond forming reactions in organic synthesis because of its atom economy and potential application in the synthesis of biologically active molecules. In this reaction, an amine, two carbonyl compounds, and acid (or base) catalysts are used to produce β-amino carbonyl compounds, which are part of various pharmaceuticals, natural products and versatile synthetic intermediates [16,17]. Due to their wide application, many efficient approaches to these compounds have been developed. In continuation of our research program [18,19] studying the synthesis of the novel heterocyclic compound which may be biologically active, we report herein the synthesis of some novel heterocyclic compounds incorporating a combination of coumarin, dicyclohexylamine and β-amino carbonyl pharmacophores.

**Scheme 1:**
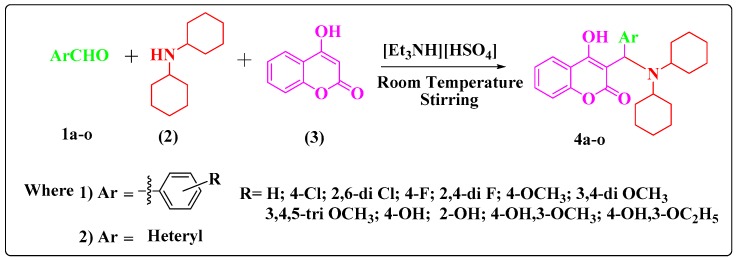
One-pot, three component synthesis of novel 3-((dicyclohexylamino)(substituted phenyl/heteryl)methyl)-4-hydroxy-2*H*-chromen-2-one derivatives **4a**–**o**.

Considering the focus on green synthesis in recent years, ionic liquids have attracted the attention of many researchers. Ionic liquids have been referred to as “designer solvents/green solvents” because their physical and chemical properties can be adjusted by varying the cation and anion. Taking into consideration the above mentioned points we have carried out the synthesis of coumarin-dicyclohexyl coupled hybrid derivatives **4a**–**o** using triethylammonium hydrogen sulphate [Et_3_NH][HSO_4_] as a solvent and easily recoverable green catalyst, as shown in Scheme 1.

All the synthesized compounds **4a**–**o** were screened for their *in vitro* antifungal and antibacterial activity. Minimum inhibitory concentration (MIC) values were determined using the standard agar method as per CLSI guidelines [20,21,22,23].

Molecular docking is a well-known computational method of drug discovery which can be used to mockup the interaction between a ligand and a protein at the atomic level and predict the behavior of ligand in the binding site of target proteins [24].

The main function of antifungal agents is to prevent the synthesis of ergosterol which is a key element of fungal plasma membrane. The enzyme lanosterol 14α-demethylase plays an important role in ergosterol synthesis in fungi [25]. Exposure of fungi to lanosterol 14α-demethylase inhibitors causes reduction of ergosterol levels. A reduction in the formation of ergosterol in the cell membrane of fungi disturbs the structure of the cell membrane and its nutrient transport and chitin synthesis functions. The net result is an inhibition of fungal growth. Most fungi cannot survive without ergosterol, so the enzymes that participate in its formation have become important targets for the drug discovery. Hence, the ergosterol extraction and quantitation assay method was performed to study the mode of action of the most potent antifungal compound **4k**. A docking study of the most potent synthesized compounds into a homology model of cytochrome P450 lanosterol 14α-demethylase of *C. albicans* was also performed [26].

To find out the drug like properties of the synthesized compounds, the physicochemical parameters based on the Lipinski RO5 (Rule of Five) [27] and Jogerson’s rule of three were determined by QikProp v3.5. This physicochemical parameter investigation of the compounds may support the pharmaceutical scientist to select the effective compounds for the development of better drugs. *In vitro* cytotoxicity studies and *in vivo* acute oral toxicity examination also suggest that the synthesized compounds are non-toxic in nature.

## 2. Result and Discussion

### 2.1. Chemistry

We report the one-pot synthesis of fifteen novel 3-((dicyclohexylamino)(substituted phenyl/heteryl)methyl)-4-hydroxy-2*H*-chromen-2-one derivatives **4a**–**o** from three component reactions of an suitable aldehyde **1a**–**o**, dicyclohexylamine (**2**) and 4-hydroxycoumarin (**3**) in the presence of [Et_3_NH][HSO_4_] as a solvent and catalyst as shown in Scheme 1.

In search of an efficient catalyst and the best experimental reaction conditions, the reaction of benzaldehyde (**1a**), dicyclohexylamine (**2**) and 4-hydroxycoumarin (**3**) at room temperature to obtain compound **4a** was considered as a standard model reaction.

Initially, the reaction was carried out in the absence of the catalyst; the product formed was in a trace/negligible amount as shown in the Supplementary Material (Table S1, entry 1). To determine the appropriate concentration of the catalyst [Et_3_NH][HSO_4_], we investigated the model reaction at different concentrations of [Et_3_NH][HSO_4_], such as 5, 10, 15, 20 and 25 mol%, where upon the product **4a** was formed in 72%, 85%, 90%, 92% and 92% yields, respectively.

The increase in concentration of the catalyst from 20 to 25 mol% did not increase the yield of the product. This indicates that 20 mol% of [Et_3_NH][HSO_4_] is sufficient for the reaction by considering the product yield. The re-usability of the ionic liquid [Et_3_NH][HSO_4_] was also studied and the results obtained are as shown in the Supplementary Material (Table S2).

With these optimized reaction conditions for the model reaction **4a**, i.e., 20 mol% [Et_3_NH][HSO_4_] catalyst, at room temperature and solvent-free conditions, we have synthesized fifteen novel 3-((dicyclohexylamino)(substituted phenyl/heteryl)methyl)-4-hydroxy-2*H*-chromen-2-one derivatives **4a**–**o**. All the synthesized compounds were characterized by IR, ^1^H-NMR, ^13^C-NMR, mass spectroscopy and elemental analyses.

### 2.2. Biological Activity

#### 2.2.1. *In Vitro* Antifungal Activity

The newly synthesized 3-((dicyclohexylamino)(substituted phenyl/heteryl)methyl)-4-hydroxy-2*H*-chromen-2-one derivatives **4a**–**o** were screened for their *in vitro* antifungal activity against different yeast and filamentous fungal pathogens. Minimum inhibitory concentration (MIC) values for *in vitro* antifungal activity were determined by the standard agar method using Miconazole as standard drug. Dimethyl sulfoxide (DMSO) was used as solvent control. The MIC (μg/mL) of all the tested compounds and that of the reference drug Miconazole has been listed in Table 1. Results from Table 1 indicated that all the synthesized compounds show good to moderate antifungal activity against the tested fungal strains. The compounds **4b**, **4c**, **4d** and **4e** having electron withdrawing groups on the phenyl ring exhibited good *in vitro* antifungal activity against the three fungal strains *Aspergillus fumigates* (NCIM-902), *Aspergillus flavus* (NCIM-539) and *Aspergillus niger* (NCIM-1196). Gratifyingly, the compound **4l** bearing a 4-hydroxy-3-ethoxy group on the phenyl ring was the most active compound in this series, exhibiting potent *in vitro* antifungal activity with MIC values of 25 µg/mL for *C. albicans*, 28 µg/mL for *C. glabrata*, 28 µg/mL for *F. oxysporum*, 36 µg/mL for *A. fumigates*, 15 µg/mL for *A. flavus*, 12 µg/mL for *A. niger* and 12 µg/mL for *C. neoformans*. Compound **4k** bearing a 4-hydroxy-3-methoxy group on the phenyl ring had MIC values of 25 µg/mL for *C. albicans*, 30 µg/mL for *C. glabrata*, 28 µg/mL for *F. oxysporum*, 38 µg/mL for *A. fumigates*, 12 µg/mL for *A. flavus*, 15 µg/mL for *A. niger* and 15 µg/mL for *C. neoformans*. Compound **4e** appeared to be equipotent *in vitro* antifungal activity when compared with the standard drug Miconazole against the fungal strains *A. fumigates*, *A. flavus*, *A. niger* and *C. neoformans*. The compounds **4m**, **4n** and **4o** were found to be the less active antifungal agents among the synthesized series.

#### 2.2.2. *In Vitro* Antibacterial Activity

The newly synthesized compounds **4a**–**o** were screened for their *in vitro* antibacterial activity against the three different bacterial strains. Minimum inhibitory concentration (MIC) values for antibacterial activity were determined by standard agar method using Ampicillin as standard drug. Dimethyl sulfoxide (DMSO) was used as solvent control. It is evident from the *in vitro* antibacterial activity results that the synthesized compounds possessed moderate to potent antibacterial activity against *Escherichia coli*, *Bacillus subtilis* and *Staphylococcus aureus* as shown in Table 2. Compound **4e** bearing a 2,4-difluoro group on the phenyl ring was 48 µg/mL for *E. coli*, 50 µg/mL for *B. subtilis* and 52 µg/mL for *S. aureus*. Compound **4c** bearing a 2,6-dichloro group on the phenyl ring was found to be the second most active antibacterial compound among the synthesized series, having MIC values of 50 µg/mL for *E. coli*, 48 µg/mL for *B. subtilis* and 50 µg/mL for *S. aureus*. The compounds **4m**, **4n** and **4o** were found to be less active antibacterial agents among the synthesized series.

Structure activity relationship (SAR) revealed that the coumarin, dicyclohexylamine and β-aminocarbonyl pharmacophore scaffolds are responsible for antimicrobial activity. From the structure activity relationships (SARs), it could be inferred in general that the modifications on the phenyl ring or replacement of phenyl ring with other heterocyclic ring significantly influence the antimicrobial activity. From the MIC values as shown in Table 1, it could be easily inferred in general that electron donating groups on phenyl ring with coumarin, dicyclohexylamine and β-aminocarbonyl pharmacophores augmented the antifungal activity.

From the antibacterial activity, it is demonstrated that the compounds with electron-withdrawing groups such as 2,4-difluoro (**4e**), 4-fluoro (**4d**), 2,6-dichloro (**4c**) and 4-chloro (**4b**) on the phenyl ring with coumarin, dicyclohexylamine and β-aminocarbonyl pharmacophores augmented the antibacterial activity. In general, the antibacterial activity decreases with phenyl ring substituent in the order of 2,4-difluro > 2,6-dichloro > 4-fluoro > 4-chloro > 4-hydroxy-3-ethoxy > 4-hydroxy-3-methoxy > 3,4-dimethoxy > 4-methoxy > 4-hydroxy > 3,4,5-trimethoxy > 2-hydroxy. Replacement of the phenyl ring with heterocyclic rings such as a pyridine ring in compound **4m**, with a thiophene ring in compound **4n** and with a furan ring as in **4o** decreased the antimicrobial activity and these compounds were found to have least antimicrobial activity.

#### 2.2.3. Ergosterol Extraction and Quantitation Assay

**Figure 1:**
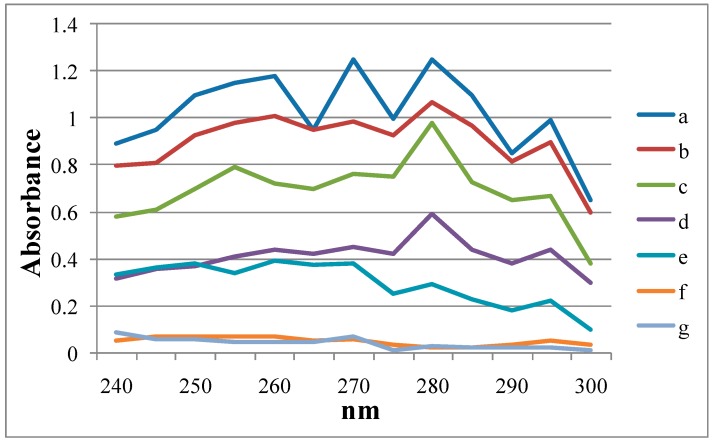
UV spectrophotometric sterol profile of *C. albicans* (NCIM3471) treated with, 0 (curve a), 1.5 (curve b), 3.1 (curve c), 6.2 (curve d), 12.5 (curve e) and 25 (curve f) µg/mL of synthesized compound **4k** and 25 (curve g) µg/mL of Miconazole. Sterols were extracted from cells and spectral profiles between 240 and 300 nm were determined.

Considering ergosterol as an important fungal cell membrane lipid, changes in its biosynthetic pathway may also cause damage to the fungal cell, preventing growth in a way similar to azole compounds such as Miconazole, Fluconazole, etc. [28]. To reveal the antifungal mechanism of the most potent synthesized compound **4k**, its influence on the sterol composition on the *C. albicans* membrane was monitored by analyzing the changes in the sterol composition in the cells of *C. albicans* by U.V. analysis. The assay was performed at various concentrations of the most potent synthesized compound **4k** such as MIC/16, MIC/8, MIC/4, MIC/2 and MIC value to quantify the content of sterol produced by *C. albicans* as shown in Figure 1.

“Curve a” in Figure 1 represents negative control (no compound). The absorption of sterols extracted from fungal culture at the wavelengths of 230 nm, and 281.5 nm was analyzed and the results obtained is as shown in Figure 1 for the most potent synthesized compound **4k**. Ergosterol and an intermediate of the metabolic pathway of ergosterol—24(28)dehydroergosterol (DHE)—absorb energy at 281.5 nm, but DHE alone shows an intense absorption at 230 nm. Changes in this pattern of absorption are indicative of interference in the synthetic pathway of ergosterol [29]. There was a change in the absorption pattern for the synthesized compound **4k** as shown in Figure 1 which proves that the synthesized compound **4k** inhibits ergosterol biosynthesis by inhibiting enzyme cytochrome P450 lanosterol 14α-demethylase of *C. albicans*. “Curve a” shows an intense ergosterol peak at 281.5 nm indicating presences of ergosterol. “Curve g” in Figure 1 represents the inhibition of ergosterol by Miconazole (standard drug) at its MIC value. As the concentration of synthesized compound **4k** increases the intensity of the ergosterol peak at 281.5 nm decreases, which indicates the decrease in concentration of ergosterol in the culture medium. At the MIC value of the synthesized compound **4k** there is almost a flat curve similar to that of Miconazole at its MIC value as shown in Figure 1. These results suggest that the compound **4k** might inhibit fungal lanosterol 14α-demethylaseby a mechanism similar to that accepted for Miconazole.

### 2.3. Computational Studies

#### 2.3.1. Molecular Docking

Docking is an effective and reliable approach to simulate the probable binding mode of ligands and proteins. Recently, coumarin derivatives were reported to inhibit cytochrome P450 lanosterol 14α-demethylase (CYP51) [30]. Based on literature reports [31] and the antifungal activity exhibited by the synthesized compounds, molecular docking studies were performed in the active site of cytochrome P450 lanosterol 14α-demethylase enzyme of *C. albicans* [26] to understand its mechanism of action. *In silico* approaches like molecular docking have become very beneficial to identify the potential targets for different ligands and are associated with the thermodynamic interactions with the target enzyme governing the inhibition of the target microorganism.

**Figure 2:**
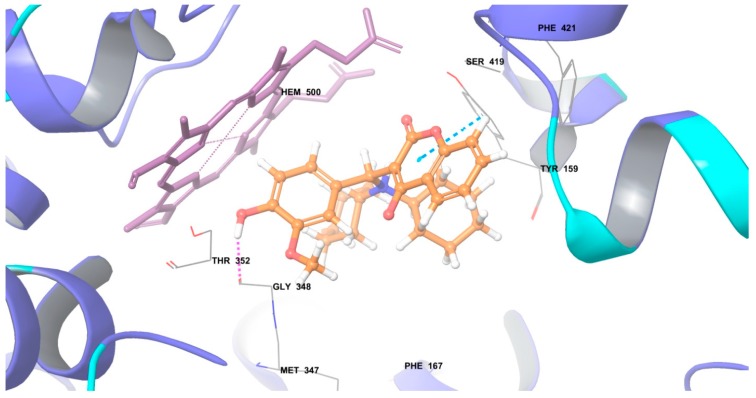
Docking pose of the compound **4k** in the active site of cytochrome P450 lanosterol 14α-demethylase of *C. albicans*.

**Figure 3:**
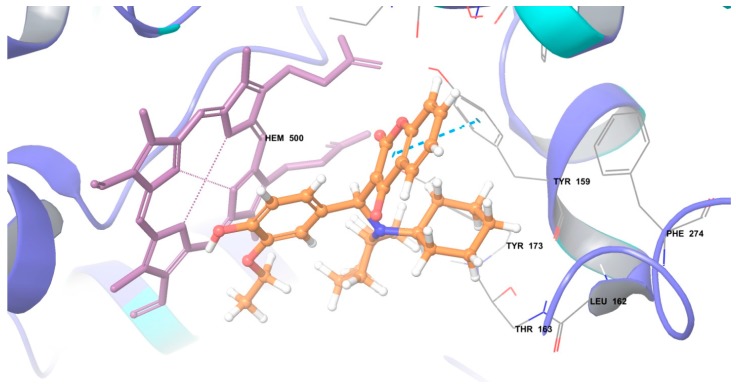
Docking pose of the compound **4l** in the active site of cytochrome P450 lanosterol 14α-demethylase of *C. albicans*.

Visual inspection of the ensuing docked structures show that all the synthesized coumarin derivatives could snugly fit into the active site of cytochrome P450 lanosterol 14α-demethylase enzyme of *C. albicans* by combination of various hydrogen and hydrophobic interactions with the enzyme. The docking scores for the most active compounds **4k** and **4l** were found to be −7.15 and −7.01 respectively. The more negative value of docking score indicates a good binding affinity of the ligand towards the target and vice versa. It is also noted that the “enzyme-ligand” complex is stabilized by hydrogen bonds and π-π interaction involving amino acid like Gly348 and Tyr159. The coumarin scaffold present in the synthesized compounds **4k** and **4l** forms a π-π interaction with the Tyr159 amino acid as shown in Figure 2 and Figure 3, respectively. The compound **4k** shows hydrogen bonding between hydroxyl group of the structure and carbonyl group of amino acid residue of Gly348 as shown in Figure 2. The docking score of the most active compound **4k** i.e., −7.15 was found to be close to that of Miconazole i.e., −7.31, the standard drug which acts by inhibiting cytochrome P450 lanosterol 14α-demethylase enzyme. Even the minimum energy for the formation of complex between ligand and receptor (glide energy) for compounds **4k** and **4l** was observed to be negative i.e., −17.06 and −18.27 kcal/mol, respectively, which as strongly suggests that the synthesized molecule could serve as a pertinent starting point for the rational design of drugs targeting cytochrome P450 lanosterol 14α-demethylase enzyme of *C. albicans.* The above findings suggest that the newly synthesized coumarin derivatives might also act by the same mechanism.

#### 2.3.2. *In Silico* ADMET Investigation Results

Table 3 shows a clear view of drug-likeness properties for the synthesized compounds **4a**–**o**. The physicochemical parameter values of the synthesized compounds **4a**–**o** obey the Lipinski RO5 (Rule of Five) without any violation, indicating that the synthesized compounds **4a**–**o** could be orally active drugs in human. It was observed that the compounds exhibited a good percent absorption (% ABS) ranging from 84.9% to 100% and were found to be non-toxic in nature.

A parameter used to evaluate aqueous solubility is Log S (S in mol/L). All the compounds have solubility values between −6.1 to −4.3 mol/L. The efficiency and distribution of a drug may be affected by the degree to which it binds to the proteins within blood plasma. Log Khsa is used for the prediction of binding to human serum albumin. The compounds showed Log Khsa values between 0.7 to 1.1 which indicate that a significant proportion of the compounds are likely to circulate freely in the blood stream and hence reach the drug target sites. The HERG K^+^ channel, which is the best known for its contribution to the electrical activity of the heart that coordinates the heart's beating, appears to be the molecular target responsible for the cardiac toxicity of a wide range of therapeutic drugs [32]. Thus, HERG K^+^ channel blockers are potentially toxic and the predicted IC_50_ values often provide reasonable predictions for cardiac toxicity of drugs in the early stages of drug discovery [33]. None of the synthesized compounds **4a**–**o** was toxic in nature as shown in Table 3. All synthesized compounds **4a**–**o** had good percent of absorption. The results of this *in silico* ADMET prediction analysis suggest that the synthesized compounds **4a**–**o** follow the criteria for orally active drugs and thus represent a pharmacologically active framework that should be considered on progressing further potential hits.

### 2.4. Toxicity Study

The toxicity study of the synthesized compounds at the early stage of research simplifies the path to clinical trials and reduces the failure of potential therapeutics at later stages of testing. Therefore, the *in vitro* cytotoxicity study, *in vivo* acute oral toxicity study and behavioral study of the most promising synthesized compounds **4e** and **4k** was evaluated.

#### 2.4.1. *In Vitro* Cytotoxicity Study

To examine the safety of the synthesized compounds, the compounds **4e** and **4k** were evaluated for their toxicity against the HeLa human cervical cancer cell line by a Sulforhodamine B (SRB) assay using Adriamycin as a standard drug [34]. The cytotoxic effect of the synthesized compounds **4e** and **4k** was checked on the cancer cell line using a concentration range between 50 and 0.78 μg/mL to determine the 50% growth inhibition, (GI_50_) value. The observed results are summarized in Table 4. The results indicated that, in SRB cytotoxicity studies, the most active compounds **4e** and **4k** can be considered as antimicrobial leads owing to their lack of significant cell toxicity against HeLa at the maximum concentration evaluated.

**Figure 4:**
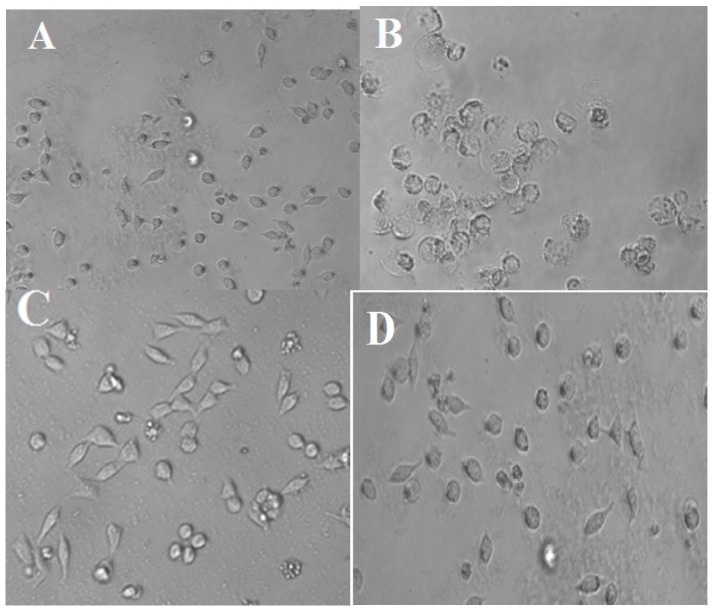
Images of *in vitro* cytotoxicity screening against the human cervical cancer cell line HeLa for Control (**A**), Positive control (**B**), Compound **4e** (**C**) at 50 µg/mL concentration, Compound **4k** (**D**) at 50 µg/mL concentration.

Figure 4 shows that the cell inhibition did not take place even up to 50 µg/mL concentration of the synthesized compounds **4e** and **4k** and hence they are not cytotoxic in nature. The disparity between the cytotoxicity and the antimicrobial activities of the synthesized compounds **4e** and **4k** suggests that these compounds exhibit high *in vitro* antimicrobial activities at non-cytotoxic concentrations.

#### 2.4.2. *In vivo* Acute Oral Toxicity Study and Behavioral Study

Animals treated with the newly synthesized compounds **4e** and **4k** were free of any toxicity as per acceptable range given by the OECD guideline No. 425 and no mortality was found up to 2000 mg/kg, which indicates that the lethal dose of the compounds is above 2000 mg/kg body weight in mice and that the compounds can be considered to be safe and could be developed in the future as good antimicrobial agents. *In vivo* acute oral toxicity study and gross behavioral studies of the newly synthesized compounds **4e** and **4k** is shown in Table 5.

## 3. Materials and Methods

### 3.1. General Information

All the reactions were performed in oven-dried glassware. All the reagents and solvents were used as obtained from the supplier or recrystallized/redistilled unless otherwise noted. The purity of the synthesized compounds was monitored by ascending thin layer chromatography (TLC) on silica gel-G coated aluminum plates (Merck, Darmstadt, Germany), visualized by iodine vapor. Melting points were determined in open capillary tubes. The FTIR spectra were obtained using a FTIR-4000instrument (JASCO, Tokyo, Japan) and peaks were expressed in terms of wave- number (cm^−1^). The ^1^H-NMR and ^13^C-NMR spectra of the synthesized compounds were recorded in CDCl_3_ at 400/100 MHz on an Avance 400 NMR Spectrometer II (Bruker, Biospin AG Industriestrasse 26, CH-8117, Fallanden, Switzerland) and using TMS as internal standard (chemical shift δ in ppm), Mass spectra were scanned on a Micromass Q-Tof system (Waters, UK). Elemental analyses (C, H and N) were done with a FLASHEA 112 analyzer (Shimadzu, Mumbai, Maharashtra, India) and all the analyses were consistent (within 0.4%) with the theoretical values.

### 3.2. Synthesis of 3-((Dicyclohexylamino)(substituted phenyl/heteryl)methyl)-4-hydroxy-2H-chromen-2-one Derivatives ***4a**–**o***

A mixture of a suitable aldehyde **1a**–**o** (1.25 mmol), dicyclohexyamine (**2**) (1.25 mmol), 4-hydroxy-coumarin (**3**) (1.25 mmol), and 20 mol% of [Et_3_NH][HSO_4_] as catalyst were taken in a 25 mL of beaker and the reaction mixture was stirred at room temperature. After completion of the reaction (monitored by TLC), the mixture was poured into ice cold water. The product obtained, was filtered and dried. The corresponding product was obtained in high purity after recrystallization of the crude product from ethanol. The authenticity of the synthesized compounds was established by ^1^H-NMR, ^13^C-NMR, IR, Mass spectra and elemental analyses.

*3-((Dicyclohexylamino)(phenyl)methyl)-4-hydroxy-2H-chromen-2-one* (**4a**), Yield 92%; m.p.: 112–114 °C; IR (KBr *v*_max_ in cm^−1^): 3500.11 (–OH of coumarin ring stretching), 3160.41(CH stretching of aromatic), 2850.55 (CH_2_ stretching of cyclohexyl ring), 1708.62 (C–O stretching), 1646.91 (C=O stretching), 1240.45 (C–N–C stretching); ^1^H-NMR δ ppm: 1.17–2.59 (m, 22 H, cyclohexyl ring), 6.07 (s, 1H, CH), 7.32–7.36 (m, 2H, coumarin ring), 7.38–7.58 (m, 5H, aromatic ring), 7.61 (t, *J* = 8.62 Hz, 1H, coumarin ring), 7.98 (d, *J* = 2.93 Hz, 1H, coumarin ring), 15.17 (s, 1H, OH); ^13^C-NMR δ ppm: 24.70, 25.39, 30.66, 56.86, 57.38, 112.82, 112.85, 114.91, 117.13, 128.77, 128.91, 129.10, 129.90, 132.66, 152.60, 164.41, 168.11; MS *m*/*z* 432.25 [M + 1]; Anal. Calcd. For C_28_H_33_NO_3_: C, 77.93; H, 7.71; N, 3.25. Found: C, 77.95; H, 7.75; N, 3.22.

*3-((4-Chlorophenyl)(dicyclohexylamino)methyl)-4-hydroxy-2H-chromen-2-one* (**4b**), Yield 95%; m.p.: 120–122 °C; IR (KBr *v*_max_ in cm^−1^): 3500.09 (–OH of coumarin ring stretching), 3162.41 (CH stretching of aromatic), 2850.77 (CH_2_ stretching of cyclohexyl ring), 1700.62 (C–O stretching), 1646.77 (C=O stretching), 1240.45 (C–N–C stretching), 740.55 (C-Cl of aromatic ring); ^1^H-NMR δ ppm: 1.19–2.56 (m, 22 H, cyclohexyl ring), 6.33 (s, 1H, CH), 7.11–7.20 (m, 4H, aromatic ring), 7.42–7.93 (m, 4H, coumarin ring), 17.38 (s, 1H, OH); ^13^C-NMR δ ppm: 24.18, 24.81, 29.13, 35.98, 52.81, 103.37, 115.37, 120.11, 122.65, 124.32, 127.49, 128.46, 129.87, 130.60, 141.13, 152.71, 165.34, 168.39; MS *m*/*z* 467.21 [M + 2]; Anal. Calcd. For C_28_H_32_ClNO_3_: C, 72.17; H, 6.92; Cl, 7.61; N, 3.01. Found: C, 72.20; H, 6.95; Cl, 7.65; N, 3.00.

*3-((2,6-Dichlorophenyl)(dicyclohexylamino)methyl)-4-hydroxy-2H-chromen-2-one* (**4c**), Yield 90%; m.p.: 126–128 °C; IR (KBr *v*_max_ in cm^−1^): 3500.09 (–OH of coumarin ring stretching), 3162.33 (CH stretching of aromatic), 2850.72 (CH_2_ stretching of cyclohexyl ring), 1707.12 (C–O stretching), 1645.77 (C=O stretching), 1242.40 (C–N–C stretching), 744.55 (C-Cl of aromatic ring); ^1^H-NMR δ ppm: 1.17–2.59 (m, 22 H, cyclohexyl ring), 6.07 (s, 1H, CH), 7.32–7.36 (m, 2H, coumarin ring), 7.38–7.54 (m, 3H, aromatic ring), 7.61 (t, *J* = 8.62 Hz, 1H, coumarin ring), 7.98 (d, *J* = 2.93 Hz, 1H, coumarin ring), 17.17 (s, 1H, OH); ^13^C-NMR δ ppm: 24.70, 25.43, 30.67, 54.57, 56.88, 112.85, 114.85, 114.91, 117.13, 128.75, 129.99, 130.81, 132.66, 133.92, 137.35, 152.60, 164.43, 168.12; MS *m*/*z* 500.17 [M + 2]; Anal. Calcd. For C_28_H_31_Cl_2_NO_3_: C, 67.20; H, 6.24; Cl, 14.17; N, 2.80. Found: C, 67.23; H, 6.28; Cl, 14.19; N, 2.78.

*3-((Dicyclohexylamino)(4-fluorophenyl)methyl)-4-hydroxy-2H-chromen-2-one* (**4d**), Yield 92%; m.p.: 122–124 °C; IR (KBr *v*_max_ in cm^−1^): 3500.09 (–OH of coumarin ring stretching), 3160.33 (CH stretching of aromatic), 2850.72 (CH_2_ stretching of cyclohexyl ring), 1708.32 (C–O stretching), 1648.67 (C=O stretching), 1245.10 (C–N–C stretching), 1053.44 (C–F of aromatic rings); ^1^H-NMR δ ppm: 1.13–2.60 (m, 22 H, cyclohexyl ring), 6.40 (s, 1H, CH), 7.13–7.25 (m, 4H, aromatic ring ), 7.49–7.98 (m, 4H, coumarin ring), 17.17 (s, 1H, OH); ^13^C-NMR δ ppm: 24.70, 25.39, 30.66, 56.86, 57.38, 112.82, 112.85, 114.91, 115.28, 115.48, 117.13, 127.63, 127.71, 129.90, 132.66, 135.65, 135.68, 152.60, 159.75, 161.54, 164.06, 168.66; MS *m*/*z* 449.24 [M + 1]; Anal. Calcd. For C_28_H_32_FNO_3_: C, 74.81; H, 7.17; F, 4.23; N, 3.12. Found: C, 74.83; H, 7.19; F, 4.25; N, 3.10.

*3-((Dicyclohexylamino)(2,4-difluorophenyl)methyl)-4-hydroxy-2H-chromen-2-one* (**4e**), Yield 89%; m.p.: 122–124 °C; IR (KBr *v*_max_ in cm^−1^): 3500.00 (–OH of coumarin ring stretching), 3166.33 (CH stretching of aromatic), 2850.72 (CH_2_ stretching of cyclohexyl ring), 1708.32 (C–O stretching), 1648.67 (C=O stretching), 1242.16 (C–N–C stretching), 1055.64 (C–F of aromatic rings); ^1^H-NMR δ ppm: 1.12–2.60 (m, 22 H, cyclohexyl ring), 6.10 (s, 1H, CH), 6.63–7.15 (m, 3H, aromatic ring), 7.45–7.96 (m, 4H, coumarin ring), 15.75 (s, 1H, OH); ^13^C-NMR δ ppm: 25.45, 26.49, 36.33, 54.44, 54.56, 56.90, 103.17, 103.37, 103.57, 111.54, 115.67, 111.88, 112.44, 112.87, 114.91, 115.19, 115.30, 117.13, 126.43, 126.70, 126.89, 129.77, 130.11, 134.65, 152.60, 159.89, 159.99, 160.27, 161.33, 161.52, 162.41, 168.59; MS *m*/*z* 467.23 [M + 1]; Anal. Calcd. For C_28_H_31_F_2_NO_3_: C, 71.93; H, 6.68; F, 8.13; N, 3.00. Found: C, 71.98; H, 6.69; F, 8.15; N, 3.27.

*3-((Dicyclohexylamino)(4-methoxyphenyl)methyl)-4-hydroxy-2H-chromen-2-one* (**4f**), Yield 86%; m.p.: 133–135 °C; IR (KBr *v*_max_ in cm^−1^): 3500.09 (–OH of coumarin ring stretching), 3162.33 (CH stretching of aromatic), 2848.92 (CH_2_ stretching of cyclohexyl ring), 1707.32 (C–O stretching), 1650.67 (C=O stretching), 1245.00 (C–N–C stretching), 1230.23 (C–OCH_3_ of aromatic rings); ^1^H-NMR δ ppm: 1.12–2.97 (m, 22 H, cyclohexyl ring), 3.87 (s, 3H, OCH_3_), 6.20 (s, 1H, CH), 6.70–7.01 (m, 4H, aromatic ring), 7.20–8.27 (m, 4H, coumarin ring), 17.60 (s, 1H, OH); ^13^C-NMR δ ppm: 24.38, 24.93, 29.36, 29.52, 35.68, 52.94, 54.89, 103.96, 113.03, 115.38, 120.30, 122.66, 124.45, 127.75, 130.51, 133.84, 152.73, 156.96, 165.88, 168.70; MS *m*/*z* 462.25 [M + 1]; Anal. Calcd. For C_29_H_35_NO_4_: C, 75.46; H, 7.64; N, 3.03. Found: C, 75.48, H, 7.66, N, 3.00.

*3-((Dicyclohexylamino)(3,4-dimethoxyphenyl)methyl)-4-hydroxy-2H-chromen-2-one* (**4g**), Yield 84%; m.p.: 138–140 °C; IR (KBr *v*_max_ in cm^−1^): 3501.00 (–OH of coumarin ring stretching), 3160.53 (CH stretching of aromatic), 2848.92 (CH_2_ stretching of cyclohexyl ring), 1709.52 (C–O stretching), 1655.17 (C=O stretching), 1245.00 (C–N–C stretching), 1235.03 (C–OCH_3_ of aromatic rings); ^1^H-NMR δ ppm: 1.13–2.59 (m, 22 H, cyclohexyl ring), 3.55 (s, 6H, OCH_3_), 6.50 (s, 1H, CH), 6.68–7.00 (m, 3H, aromatic ring), 7.35–7.96 (m, 4H, coumarin ring), 15.17 (s, 1H, OH); ^13^C-NMR δ ppm: 25.55, 26.99, 31.23, 56.05, 56.84, 59.00, 111.02, 112.82, 112.85, 113.02, 114.91, 117.31, 122.71, 128.78, 129.91, 132.66, 148.81, 152.66, 164.45, 168.23; MS *m*/*z* 491.27 [M + 1]; Anal. Calcd. For C_30_H_37_NO_5_: C, 73.29; H, 7.59; N, 2.85. Found: C, 73.32; H, 7.61; N, 2.82.

*3-((Dicyclohexylamino)(3,4,5-trimethoxyphenyl)methyl)-4-hydroxy-2H-chromen-2-one* (**4h**), Yield 82%; m.p.: 136–138 °C; IR (KBr *v*_max_ in cm^−1^): 3500.09 (–OH of coumarin ring stretching), 3168.03 (CH stretching of aromatic), 2850.02 (CH_2_ stretching of cyclohexyl ring), 1710.02 (C–O stretching), 1658.07 (C=O stretching), 1244.90 (C–N–C stretching), 1234.93 (C–OCH_3_ of aromatic rings); ^1^H-NMR δ ppm: 1.14–2.60 (m, 22 H, cyclohexyl ring), 3.56 (s, 9H, OCH_3_), 6.01 (s, 1H, CH), 6.29 (s, 2H, aromatic ring), 7.42–7.89 (m, 4H, coumarin ring), 15.17 (s, 1H, OH); ^13^C-NMR δ ppm: 24.79, 25.66, 30.73, 56.65, 59.34, 62.56, 108.22, 112.82, 112.85, 114.91, 116.92, 129.95, 131.77, 132.66, 138.78, 152.66, 153.11, 162.09, 168.75; MS *m*/*z* 521.28 [M + 1]; Anal. Calcd. For C_31_H_39_NO_6_: C, 71.38; H, 7.54; N, 2.69. Found: C, 71.39; H, 7.57; N, 2.65.

*3-((Dicyclohexylamino)(4-hydroxyphenyl)methyl)-4-hydroxy-2H-chromen-2-one* (**4i**), Yield 90%; m.p.: 128–130 °C; IR (KBr *v*_max_ in cm^−1^): 3500.09 (–OH of coumarin ring stretching), 3333.56 (C–OH of aromatic ring), 3170.03 (CH stretching of aromatic), 2849.12 (CH_2_ stretching of cyclohexyl ring), 1715.02 (C–O stretching), 1660.07 (C=O stretching), 1245.00 (C–N–C stretching); ^1^H-NMR δ ppm: 1.14–2.60 (m, 22 H, cyclohexyl ring), 6.15 (s, 1H, CH), 6.29–6.90 (m, 4H, aromatic ring ), 7.32–7.89 (m, 4H, coumarin ring), 17.17 (s, 1H, OH); ^13^C-NMR δ ppm: 24.79, 25.66, 30.63, 56.84, 57.46 112.82, 112.92, 114.91, 116.59, 117.13, 129.95, 130.77, 132.66, 152.11, 161.09, 164.45, 168.23; MS *m*/*z* 447.24 [M + 1]; Anal. Calcd. For C_28_H_33_NO_4_: C, 75.14; H, 7.43; N, 3.13. Found: C, 75.16; H, 7.46; N, 3.10.

*3-((Dicyclohexylamino)(2-hydroxyphenyl)methyl)-4-hydroxy-2H-chromen-2-one* (**4j**), Yield 88%; m.p.: 130–132 °C; IR (KBr *v*_max_ in cm^−1^): 3500.09 (–OH of coumarin ring stretching), 3333.86 (C–OH of aromatic ring), 3172.03 (CH stretching of aromatic), 2848.92 (CH_2_ stretching of cyclohexyl ring), 1720.02 (C–O stretching), 1665.00 (C=O stretching), 1243.99 (C–N–C stretching); ^1^H-NMR δ ppm: 1.14–2.62 (m, 22 H, cyclohexyl ring), 6.12 (s, 1H, CH), 6.29–6.90 (m, 4H, aromatic ring ), 7.33–7.98 (m, 4H, coumarin ring), 17.17 (s, 1H, OH); ^13^C-NMR δ ppm: 24.73, 25.65, 30.62, 50.25, 56.64, 112.85, 114.91, 115.48, 116.84, 117.12, 122.95, 126.86, 129.19, 129.90, 130.87, 132.66, 152.90, 161.19, 164.46, 168.45; MS *m*/*z* 447.25 [M + 1]; Anal. Calcd. For C_28_H_33_NO_4_: C, 75.14; H, 7.43; N, 3.13. Found: C, 75.16; H, 7.46; N, 3.11.

*3-((Dicyclohexylamino)(4-hydroxy-3-methoxyphenyl)methyl)-4-hydroxy-2H-chromen-2-one* (**4k**), Yield 86%; m.p.: 144–146 °C; IR (KBr *v*_max_ in cm^−1^): 3500.09 (–OH of coumarin ring stretching), 3334.56 (C–OH of aromatic ring), 3170.03 (CH stretching of aromatic), 2848.90 (CH_2_ stretching of cyclohexyl ring), 1725.02 (C–O stretching), 1665.10 (C=O stretching), 1245.00 (C–N–C stretching), 1234.95 (C–OCH_3_ of aromatic rings); ^1^H-NMR δ ppm: 1.11–2.37 (m, 22 H, cyclohexyl ring), 3.85 (s, 3H, OCH_3_), 5.39 (s, 1H, OH), 6.09 (s, 1H, CH), 6.78–7.31 (m, 3H, aromatic ring), 7.33–7.99 (m, 4H, coumarin ring), 17.17 (s, 1H, OH); ^13^C-NMR δ ppm: 24.70, 25.39, 30.66, 56.05, 56.86, 59.00, 110.83, 112.82, 112.85, 114.91, 115.14, 117.13, 122.68, 129.05, 129.90, 132.66, 146.45, 147.77, 152.60, 164.41, 168.22; MS *m*/*z* 478.25 [M + 1]; Anal. Calcd. For C_29_H_35_NO_5_: C, 72.93; H, 7.39; N, 2.93. Found: C, 72.96; H, 7.41; N, 2.90.

*3-((Dicyclohexylamino)(3-ethoxy-4-hydroxyphenyl)methyl)-4-hydroxy-2H-chromen-2-one* (**4l**), Yield 86%; m.p.: 140–142 °C; IR (KBr *v*_max_ in cm^−1^): 3500.11 (–OH of coumarin ring stretching), 3333.66 (C–OH of aromatic ring), 3170.03 (CH stretching of aromatic), 2848.12 (CH_2_ stretching of cyclohexyl ring), 1725.02 (C–O stretching), 1665.10 (C=O stretching), 1242.97 (C–N–C stretching); ^1^H-NMR δ ppm: 1.14–1.62 (m, 20 H, cyclohexyl ring), 1.65 (t, *J* = 7.10 Hz, 3H, OCH_2_CH_3_ ), 2.55 (m, 2H, cyclohexyl ring), 3.57 (s, 3H, OCH_3_), 4.09 (q, *J* = 7.12 Hz, 2H, OCH_2_CH_3_), 5.35 (s, 1H, OH), 6.09 (s, 1H, CH), 6.69–6.87 (m, 3H, aromatic ring ), 7.43–7.88 (m, 4H, coumarin ring), 15.18 (s, 1H, OH); ^13^C-NMR δ ppm: 14.88, 24.30, 25.65, 30.72, 56.86, 59.00, 63.96, 112.63, 112.82, 112.85, 114.91, 114.99, 117.13, 122.60, 127.14, 129.90, 132.66, 149.53, 150.15, 152.60, 164.45, 168.32; MS *m*/*z* 491.27 [M + 1]; Anal. Calcd. For C_30_H_37_NO_5_: C, 73.29; H, 7.59; N, 2.85. Found: C, 73.33; H, 7.62; N, 2.81.

*3-((Dicyclohexylamino)(pyridin-2-yl)methyl)-4-hydroxy-2H-chromen-2-one* (**4m**), Yield 84%; m.p.: 148–150 °C; IR (KBr *v*_max_ in cm^−1^): 3500.00 (–OH of coumarin ring stretching), 3170.03 (CH stretching of aromatic), 2848.90 (CH_2_ stretching of cyclohexyl ring), 1725.02 (C–O stretching), 1665.10 (C=O stretching), 1245.00 (C–N–C stretching); ^1^H-NMR δ ppm: 1.21–2.36 (m, 22 H, cyclohexyl ring), 5.07 (s, 1H, CH), 7.20 (d, *J* = 7.62 Hz, 1H, pyridine ring), 7.36–7.61 (m, 3H, coumarin ring), 7.63–7.71 (d, *J* = 7.62 Hz, 2H, pyridine ring), 7.98 (d, *J* = 7.42 Hz, 1H, coumarin ring), 8.69 (d, *J* = 5.12 Hz, 1H, pyridine ring), 15.17 (s, 1H, OH); ^13^C-NMR δ ppm: 24.70, 25.39, 30.66, 56.86, 59.65, 102.82, 112.85, 114.91, 117.13, 122.46, 126.45, 129.90, 132.66, 137.89, 148.23, 152.60, 155.44, 160.81, 164.41; MS *m*/*z* 432.25 [M + 1]; Anal. Calcd. For C_27_H_32_N_2_O_3_: C, 74.97; H, 7.46; N, 6.48. Found: C, 74.99; H, 7.48; N, 6.44.

*3-((Dicyclohexylamino)(thiophen-2-yl)methyl)-4-hydroxy-2H-chromen-2-one* (**4n**), Yield 88%; m.p.: 140–142 °C; IR (KBr *v*_max_ in cm^−1^): 3501.00 (–OH of coumarin ring stretching), 3170.03 (CH stretching of aromatic), 2848.90 (CH_2_ stretching of cyclohexyl ring), 1725.02 (C–O stretching), 1665.10 (C=O stretching), 1245.00 (C–N–C stretching); ^1^H-NMR δ ppm: 1.14–2.62 (m, 22H, cyclohexyl ring), 6.48 (s, 1H, CH), 6.65–7.06 (m, 3H, thiophene ring ), 7.18–8.14 (m, 4H, coumarin ring), 17.82 (s, 1H, OH);^13^C-NMR δ ppm: 24.13, 24.87, 29.29, 33.07, 52.47, 103.83, 115.34, 120.07, 122.29, 122.68, 122.99, 124.30, 125.93, 130.75, 148.19, 152.60, 164.52, 168.22; MS *m*/*z* 438.25 [M + 1]; Anal. Calcd. For C_26_H_31_NO_3_S: C, 71.36; H, 7.14; N, 3.20; S 7.33. Found: C, 71.38; H, 7.18; N, 3.18; S, 7.32.

*3-((Dicyclohexylamino)(furan-2-yl)methyl)-4-hydroxy-2H-chromen-2-one* (**4o**), Yield 86%; m.p.: 148–150 °C; IR (KBr *v*_max_ in cm^−1^): 3500.00 (–OH of coumarin ring stretching), 3170.03 (CH stretching of aromatic), 2848.90 (CH_2_ stretching of cyclohexyl ring), 1725.02 (C–O stretching), 1665.10 (C=O stretching), 1245.40 (C–N–C stretching); ^1^H-NMR δ ppm: 1.25–2.51 (m, 22 H, cyclohexyl ring), 5.09 (s, 1H, CH), 6.38–7.36 (m, 3H, thiophene ring ), 7.47–7.99 (m, 4H, coumarin ring), 17.17 (s, 1H, OH);^13^C-NMR δ ppm: 24.70, 25.39, 30.66, 55.62, 57.90, 104.46, 108.38, 110.53, 112.85, 114.91, 117.13, 129.90, 132.66, 144.08, 152.60, 153.45, 164.75, 168.77; MS *m*/*z* 422.25 [M + 1]; Anal. Calcd. For C_26_H_31_NO_4_: C, 74.08; H, 7.41; N, 3.32. Found: C, 74.08; H, 7.41; N, 3.32.

### 3.3. In Vitro *Antimicrobial Activity*

All the synthesized compounds were screened for their *in vitro* antifungal and antibacterial activity. The antibacterial activity was evaluated against three human pathogenic bacterial strains: *Escherichia coli* (NCIM-2256), *Bacillus subtilis* (NCIM-2063) and *Staphylococcus aureus* (NCIM-2901). The antifungal activity was evaluated against seven human pathogenic fungal strains: *Candida albicans* (NCIM-3471), *Candida glabrata* (NCYC-388), *Fusarium oxysporum* (NCIM-1332), *Aspergillus fumigates* (NCIM-902), *Aspergillus flavus* (NCIM-539), *Aspergillus niger* (NCIM-1196) and *Cryptococcus neoformans* (NCIM-576), which are often encountered clinically. Miconazole was used as standard drug. Minimum inhibitory concentration (MIC) values were determined as per CLSI guidelines [20,21,22,23]. Dimethyl sulfoxide (DMSO) was used as solvent control.

#### 3.3.1. *In Vitro* Antifungal Activity

Antifungal activity was determined as per CLSI (formerly, NCCLS) guidelines [20,21,22,23]. The synthesized compounds **4a**–**o** and the standard drug Miconazole were dissolved in DMSO solvent. The medium yeast nitrogen base was dissolved in phosphate buffer pH 7 and it was autoclaved at 110 °C for 10 min. With each set a growth control without the antifungal agent and solvent control DMSO were included. The fungal strains were freshly sub cultured on to Sabouraud dextrose agar (SDA) and incubated at 25 °C for 72 h. The fungal cells were suspended in sterile distilled water and diluted to get 10^5^ cells/mL. Ten μL of the standardized suspension was inoculated onto the control plates and the media incorporated with the antifungal agents. The inoculated plates were incubated at 25 °C for 48 h. The readings were taken at the end of 48 h and 72 h. The MIC was the lowest concentration of drug preventing growth of macroscopically visible colonies on drug containing plates when there was visible growth on the drug free control plates.

#### 3.3.2. *In Vitro* Antibacterial Activity

All the synthesized compounds **4a**–**o** were screened for their *in vitro* antibacterial activity. Minimum inhibitory concentration (MIC) values were determined using method recommended by National Committee for Clinical Laboratory Standards (NCCLS). *In vitro* antibacterial activities of the synthesized compounds **4a**–**o** were tested in Nutrient Broth (NB) for bacteria by the two fold serial dilution method. Seeded broth (broth containing microbial spores) was prepared in NB from 24 h old bacterial cultures on nutrient agar (Hi-media) at 37± 1 °C. The bacterial suspension was adjusted with sterile saline to a concentration of 1× 10^−4^–10^−5^ C.F.U. The synthesized compounds and standard drug Ampicillin were prepared by two fold serial dilutions to obtain the required concentrations of 400, 200, 100, 50, 25, 12.5, 6.25 and 3.13 μg/mL. The tubes were incubated in BOD incubators at 37± 1 °C for bacteria. The MICs were recorded by visual observations after 24 h (for bacteria) of incubation [20,21,22,23].

#### 3.3.3. Ergosterol Extraction and Quantitation Assay

A single *Candida albicans* (NCIM-3471) colony from an overnight Sabouraud dextrose agar plate culture was used to inoculate 50 mL of Sabouraud dextrose broth for control and for various concentrations of the molecules. The cultures were incubated for 16 h and harvested by centrifugation at 2700 rpm (856× *g*) for five min. The net weight of the cell pellet was determined. Three milliliters of 25% alcoholic potassium hydroxide solution was added to each pellet and vortex mixed for one min. Cell suspensions were transferred to sterile borosilicate glass screw-cap tubes and were incubated in an 85 °C water bath for one hour. Following incubation, the tubes were allowed to cool. Sterols were then extracted by addition of a mixture of one mL of sterile distilled water and 3 mL of n-heptane followed by vigorous vortex mixing for 3 min. The heptane layer was transferred to a clean borosilicate glass screw-cap tube and stored at −20 °C. Prior to analysis, 0.6 mL aliquot of sterol extract was diluted five fold in 100% ethanol and scanned spectrophotometrically between 240 nm and 300 nm with a spectrophotometer (UV-Visible Spectrophotometer 2100 Thermo Fischer Scientific, Waltham, MA, USA). The presence of ergosterol and the late sterol intermediate 24(28) dehydroergosterol (DHE) in the extracted sample resulted in a characteristic four-peaked curve. The absence of detectable ergosterol in extracts was indicated by a flat line. A dose-dependent decrease in the height of the absorbance peaks was evident and corresponded to decreased ergosterol concentration [28].

### 3.4. Computational Studies

#### 3.4.1. Molecular Docking

For the docking of ligands into the active sites of proteins and for estimating the binding affinities of the docked compounds, the 3D model structure of cytochrome P450 lanosterol 14α-demethylase of *C. albicans* was built using homology modeling. The binding interactions were studied using Maestro 9.1 using Glide v6.8 (Schrodinger, LLC, New York, NY, USA). All the compounds were built using Maestro build panel and optimized to lower energy conformers using Ligprep v3.5.9 (Schrodinger, LLC). The 3D model structure of cytochrome P450 lanosterol 14α-demethylase of *C. albicans* was built using homology modeling. Amino acid sequence of enzyme was obtained from the Universal Protein Resource (http://www.uniprot.org/) (Accession Code: P10613), and sequence homologous was obtained from Protein Data Bank (PDB) using Blast search. The procedure for homology modeling was followed as per the literature [26].

#### 3.4.2. *In Silico* ADMET Prediction

A computational study of the synthesized compounds **4a**–**o** was performed for prediction of ADMET properties. The absorption, distribution, metabolism, excretion and toxicity (ADMET) properties of all the compounds were predicted using QikProp v3.5 (2015, Schrödinger, LLC). In the present study, we have calculated the molecular volume (MV), molecular weight (MW), predicted octanol–water partition coefficient (log Po/w), number of hydrogen bond acceptors (n-ON), number of hydrogen bonds donors (n-OHNH), Percentage of human oral absorption (% ABS), Van Der Waals surface area of polar nitrogen and oxygen atoms (Polar Surface Area), Log S (water solubility), BIP_Caco−2_ (apparent Caco-2 cell permeability), Log Khsa (binding to human serum albumin) and Log HERG (toxicity study).

### 3.5. Toxicity Study

#### 3.5.1. *In Vitro* Cytotoxicity Study

To study the safety profile and to explore the selective antimicrobial activity of the most active compounds **4e** and **4k**, *in vitro* cytoxocity study was performed. This study proves that the synthesized compounds **4e** and **4k** show only antimicrobial activity at their MIC values and do not kill the human cell lines indicating their safety profile and selectivity towards antimicrobial activity. *In vitro* cytotoxicity study of the synthesized compounds **4e** and **4k** was performed against HeLa (Human cervical cancer cell line) by Sulforhodamine B (SRB) assay using Adriamycin as positive control [32]. The images were captured using an Eclipse Ti-S Inverted Research Microscope (Nikon, Tokyo, Japan). Image processing software used was NIS-Elements.

#### 3.5.2. *In Vivo* Acute Oral Toxicity Study and Behavioral Study

The *in vivo* acute oral toxicity study for the most active compounds **4e** and **4k** was carried out by the following OECD guideline no. 425 using Swiss albino mice (18–22 gm weight) quarantined at animal house at Y.B. Chavan College of Pharmacy, Aurangabad IAEC approval number CPCSEA/IAEC/P’col-52/2015-16/115. Each group consists of six mice (overnight fasted) and kept in colony cage at 25 ± 2 °C with 55% relative humidity and 12 h of light and dark cycle. A specified dose of 100, 250, 500, 750, 1000, 1500 and 2000 mg/kg body weight of mice was administered orally as a single dose. The acute toxic symptoms and the behavioral changes produced by the synthesized compounds were observed continuously for 4 h periods at 8th, 12th and 24th h on set of toxic symptoms and the gross behavioral changes were also recorded. These animals were maintained for further 10 days with observation made daily.

## 4. Conclusions

In this study, a suite of new antifungal and antibacterial agents has been synthesized using a green protocol. The *in vitro* antibacterial and antifungal activity results suggest that compounds **4b**, **4c**, **4d**, **4e**, **4k** and **4l** possess potent *in vitro* antifungal and antibacterial activity. In the present series compound **4e** with a 2,4-difluorogroup on the phenyl ring was found to be the most potent antibacterial agent. Compound **4k** with 4-hydroxy-3-methoxy group on the phenyl ring was found to be the most potent antifungal agent. The ergosterol extraction and quantitation assay method was applied to study the mode of action of the most potent compound **4k**. The assay results indicate that the synthesized compound **4k** act by the inhibition of the ergosterol biosynthesis by inhibiting lanosterol 14α-demethylaseenzyme. Molecular docking was performed to mockup the interaction between the most active compounds and the active site of lanosterol 14 α-demethylase at the atomic level, to predict the probable mechanism of action of the synthesized compounds as antifungal agent. The molecular docking results suggest that the synthesized compounds exhibit inhibitory activity against lanosterol 14 α-demethylase. Analysis of the ADMET parameters for the synthesized compounds **4a**–**o** has shown that these compounds have good oral drug like properties and could be developed as oral drug candidates. Furthermore, the *in vitro* cytotoxicity test against HeLa cells revealed that the compounds **4e** and **4k** possess a high safety profile and also indicated the selectivity of the antimicrobial action. The *in vivo* acute oral toxicity study for the newly synthesized compounds **4e** and **4k** was performed using Swiss albino mice and there were no mortalities and no significant behavioral changes observed during the first 24 h at all the tested concentrations. The synthesized compounds **4b**, **4c**, **4d**, **4e**, **4k** and **4l** are very attractive antimicrobial leads and can serve as an excellent scaffold for the further study. Therefore, we hope the results of our present analysis will assist researchers to discover and synthesize new coumarin derivatives.

## Figures and Tables

**Table 1:** *In vitro* antifungal activity of the synthesized compounds **4a**–**o**.

Compound	MIC ^a^ μg/mL						
	*Candida albicans*	*Candida glabrata*	*Fusarium oxysporum*	*Aspergillus fumigates*	*Aspergillus flavus*	*Aspergillus niger*	*Cryptococcus neoformans*
**4a**	66	58	55	84	38	43	54
**4b**	30	32	34	30	**14**	**15**	**15**
**4c**	30	28	35	28	**15**	**18**	**14**
**4d**	28	30	30	28	**15**	**20**	**18**
**4e**	28	26	30	28	**12**	**12**	**12**
**4f**	43	57	39	44	20	22	20
**4g**	50	57	35	52	24	20	26
**4h**	48	64	45	50	38	34	34
**4i**	32	35	35	42	25	28	24
**4j**	46	47	38	55	32	30	35
**4k**	**25**	**30**	**28**	**38**	**12**	**15**	**15**
**4l**	**25**	**28**	**28**	**36**	**15**	**12**	**12**
**4m**	48	46	40	45	25	22	28
**4n**	55	53	58	67	32	38	33
**4o**	56	55	55	65	46	49	48
**Miconazole**	25	25	25	35	12	12	12

^a^ Values are the average of three readings; MIC: Minimum inhibitory concentration. Bold value indicates equipotent/nearness to values of standard drug (Miconazole).

**Table 2:** *In vitro* antibacterial activity of the synthesized compounds **4a**–**o**.

Compound	MIC ^a^ μg/mL		
	*Escherichia coli*	*Bacillus subtilis*	*Staphylococcus aureus*
**4a**	70	68	65
**4b**	**52**	**50**	**54**
**4c**	**50**	**48**	**50**
**4d**	**50**	**49**	**50**
**4e**	**48**	**50**	**52**
**4f**	64	58	55
**4g**	62	60	62
**4h**	68	68	66
**4i**	64	66	67
**4j**	68	72	72
**4k**	55	55	54
**4l**	56	54	54
**4m**	68	74	78
**4n**	65	74	72
**4o**	66	74	70
Ampicillin	50	50	50

^a^ Values are the average of three readings; MIC: Minimum inhibitory concentration. Bold value indicates equipotent/nearness to values of standard drug (Ampicillin).

**Table 3:** *In silico* physicochemical pharmacokinetic parameters important for good oral bioavailability of the synthesized compounds **4a**–**o**.

Entry	M.W ^a^	Log Po/w ^b^ (−2.0 to 6.5)	n-ON ^c^ (<10)	n-OHNH ^d^ (<5)	PSA ^e^ (7–200.0)	Log Khsa ^f^ (−1.5–1.2)	Log S ^g^ (−6–0.5)	% ABS ^h^	# Meta ^i^ (1–8)	Log HERG ^j^ (below −5)	Lipinski Rule of 5 (≤1)
**4a**	431.5	4.9	5.2	1	51.7	0.9	−4.7	100	4	−6.1	0
**4b**	466.0	5.5	5.2	1	48.5	1.1	−5.5	100	3	−6.1	0
**4c**	498.1	5.2	5.2	1	48.9	1.0	−5.0	100	3	−6.0	0
**4d**	449.5	5.2	5.2	1	48.6	1.0	−5.1	100	3	−6.1	0
**4e**	467.5	5.2	5.2	1	50.7	1.0	−4.9	96.9	4	−6.0	0
**4f**	461.6	5.0	6.0	1	58.9	0.9	−4.7	95.9	4	−6.1	0
**4g**	491.6	5.2	6.7	1	64.5	1.0	−5.2	100	4	−6.2	0
**4h**	521.0	5.3	7.5	1	69.6	1.0	−5.3	84.9	6	−6.1	0
**4i**	447.5	4.2	6	1	71.8	0.8	−4.6	95.0	4	−6.2	0
**4j**	447.5	4.1	6	1	70.9	0.8	−4.6	95.0	4	−6.2	0
**4k**	478.2	4.2	6	1	71.8	0.9	−4.7	95.0	4	−6.0	0
**4l**	490.2	4.1	6	1	50.7	1.0	−5.1	96.9	4	−6.0	0
**4m**	432.5	4.4	6.2	1	62.0	0.7	−4.4	100	5	−6.2	0
**4n**	437.5	4.8	5.2	1	47.7	0.8	−4.6	100	4	−6.0	0
**4o**	421.5	4.4	5.7	1	53.5	0.7	−4.3	100	4	−6.1	0

^a^ Molecular weight of the molecule; ^b^ Predicted octanol–water partition coefficient (log Po/w) (–2.0 to 6.5); ^c^ n-ON number of hydrogen bond acceptors ≤10; ^d^ n-OHNH number of hydrogen bonds donors ≤5; ^e^ Polar surface area (PSA) (7.0–200.0); ^f^ Logarithm of predicted binding constant to human serum albumin (log Khsa) (−1.5 to 1.2); ^g^ Logarithm of Aqueous solubility(Log S) (−6 to 0.5); ^h^ Percentage human oral absorption (% ABS) (>80% is high, <25% is poor); ^i^ Metabolism (1–8); ^j^ Predicted IC_50_ value for blockage of HERG K^+^ channels (Log HERG) (below −5).

**Table 4:** *In vitro* cytotoxicity study.

Compound	GI_50_ (µg/mL) ^a^
	HeLa
**4e**	>50
**4k**	>50
Adriamycin	0.5

^a^ GI_50_ is the concentration exhibiting 50% inhibition of the growth as compared to the growth of control; HeLa (Human cervical cancer cell line); Adriamycin: standard drug.

**Table 5:** *In vivo* acute oral toxicity study and gross behavioral studies of the synthesized compounds **4e** and **4k**.

Observation	Compound	
	4e	4k
**Toxicity**	N	N
**Deaths**	N	N
**Eye Lacrimation**	N	N
**Salivation**	N	N
**Diarrhea**	N	N
**Respiration Problems**	N	N
**Straub Tail**	N	N
**Pilo Erection**	N	N
**Convulsions**	N	N
**Normal Motor Activity**	Y	Y
**Stereotypy**	N	N
**Tremors**	N	N
**Sedation**	N	N
**Hypnosis**	N	N
**Muscle Spasms**	N	N

N: no; Y: yes.

## Supplementary Materials

Supplementary Materials are available online.
