# Supplementary material for: Facile Synthesis of Novel Coumarin Derivatives, Antimicrobial Analysis, Enzyme Assay, Docking Study, ADMET Prediction and Toxicity Study
Source: Molecules. 2017 Jul 13;22(7):1172. doi: 10.3390/molecules22071172 (PMC6152127; doi:10.3390/molecules22071172)

Supplementary Material

Facile Synthesis Of Novel Coumarin Derivatives, Antimicrobial analysis, Enzyme Assay, Docking Study, ADMET Prediction And Toxicity Study.

Shailee V. Tiwari [1], Julio A. Seijas [2], Maria Pilar Vazquez-Tato [2], Aniket P. Sarkate [3], Kshipra S. Karnik[3] and Anna Pratima G. Nikalje [1]*

[1] Y.B. Chavan College of Pharmacy, Dr. Rafiq Zakaria Campus, Rauza Baug, Aurangabad, Maharashtra 431001, India; shailee2010@gmail.com

[2] Departamento de Química Orgánica, Facultad de Ciencias, Universidad of Santiago De Compostela, Alfonso X el Sabio, Lugo 27002, Spain; julioa.seijas@usc.es (J.A.S.); pilar.vazquez.tato@usc.es (M.P.V.-T.)

[3] Department of Chemical Technology, Dr. Babasaheb Ambedkar Marathwada University, Aurangabad 431004, Maharashtra, India.

* Correspondence: annapratimanikalje@gmail.com ; Tel.: +91-916-892-9111

Table S1 Effect of [Et_3NH][HSO_4] catalyst concentration on model reaction **4a**

Entry	[Et_3NH][HSO_4] mol%	Time (min)	Yield (%)
1.	No catalyst	90	Trace
2.	5	85	72
3.	10	60	85
4.	15	50	90
5.	20	30	92
6.	25	30	92

Table S2 Reusability of [Et_3NH][HSO_4] catalyst for model reaction **4a**

Entry	Run	Time	Yield
1.	1	30	92
2.	2	30	92
3.	3	30	90
4.	4	30	88
5.	5	30	88

^{1}HNMR **4b**

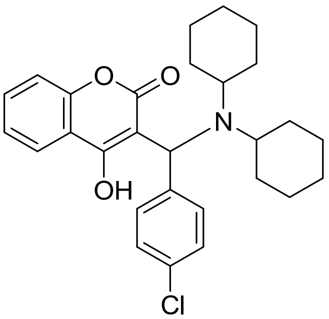

3-((4-chlorophenyl)(dicyclohexylamino)methyl)-4-hydroxy-2*H*-chromen-2-one

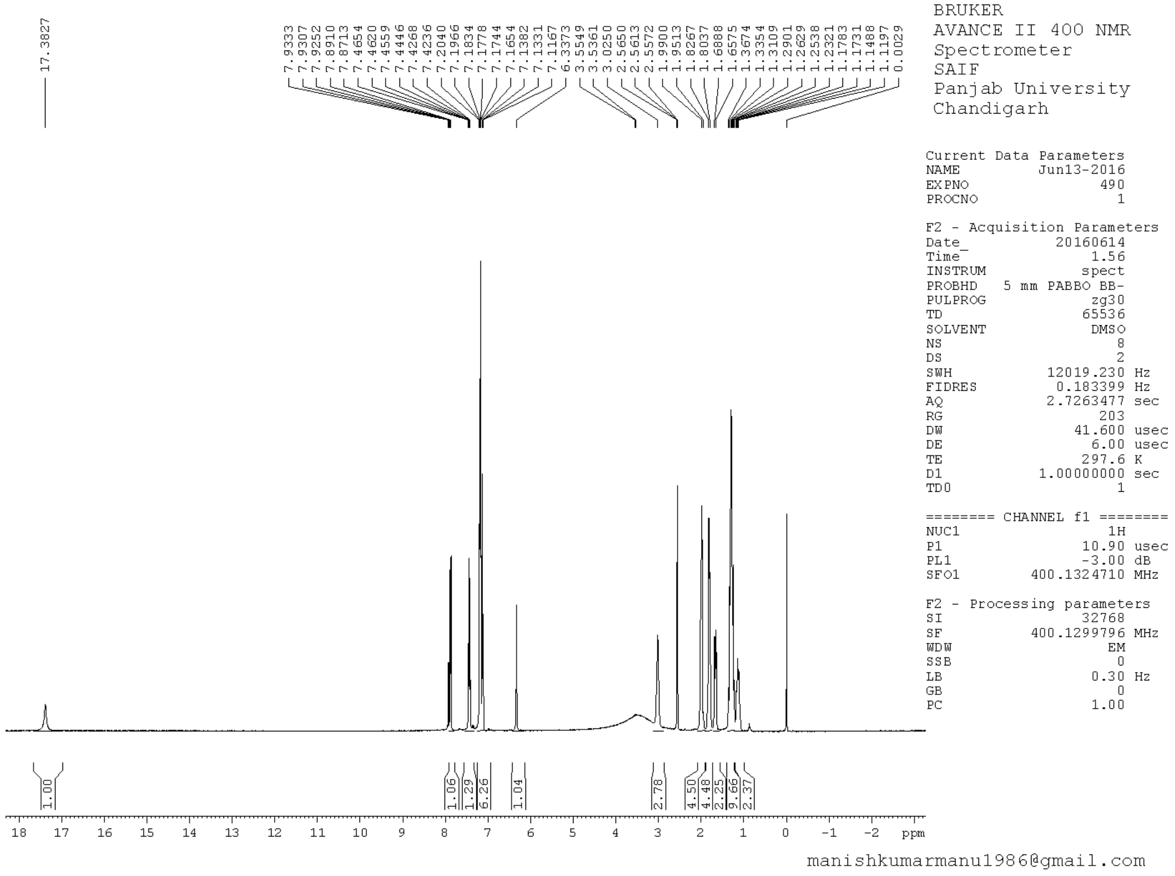

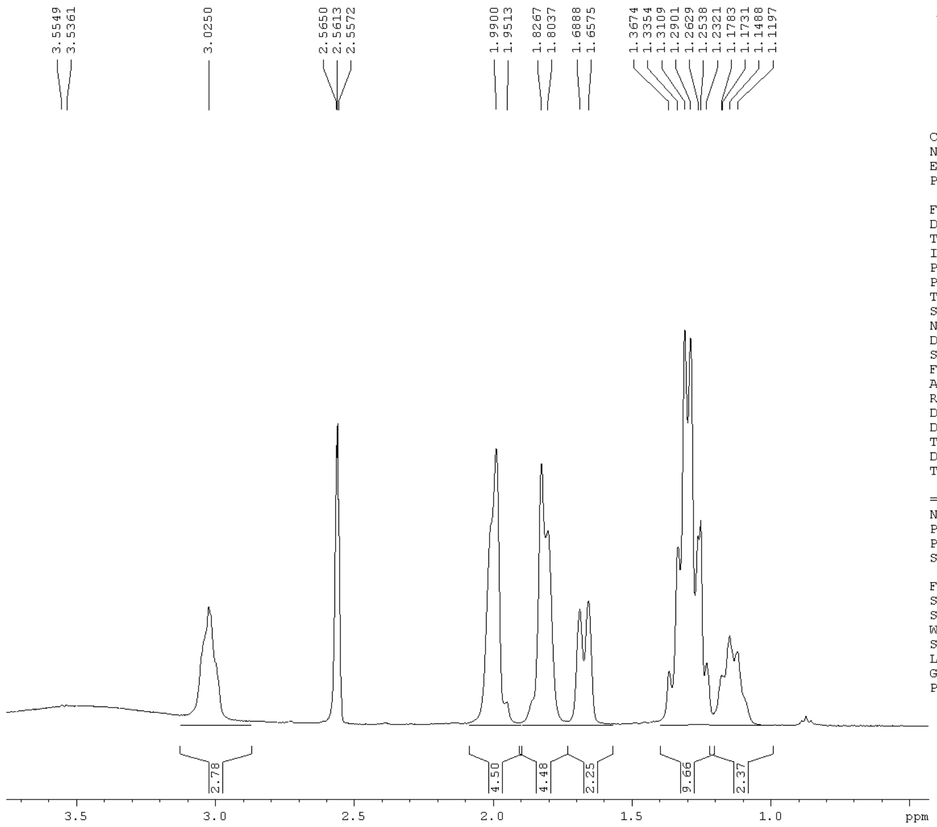

BRUKER
AVANCE II 400 NMR
Spectrometer
SAIF
Panjab University
Chandigarh

Current Data Parameters

NAME	Jun13-2016
EXPNO	490
PROCNO	1

F2 - Acquisition Parameters

Date_	20160614
Time	1.56
INSTRUM	spect
PROBHD	5 mm PABBO BB-
PULPROG	zg30
TD	65536
SOLVENT	DMSO
NS	8
DS	2
SWH	12019.230 Hz
FIDRES	0.183399 Hz
AQ	2.7263477 sec
RG	203
DW	41.600 usec
DE	6.00 usec
TE	297.6 K
D1	1.00000000 sec
TD0	1

======== CHANNEL f1 ========

NUC1	1H
P1	10.90 usec
PL1	-3.00 dB
SFO1	400.1324710 MHz

F2 - Processing parameters

SI	32768
SF	400.1299796 MHz
WDW	EM
SSB	0
LB	0.30 Hz
GB	0
PC	1.00

manishkumarmanu1986@gmail.com

^{13}CNMR **4b**

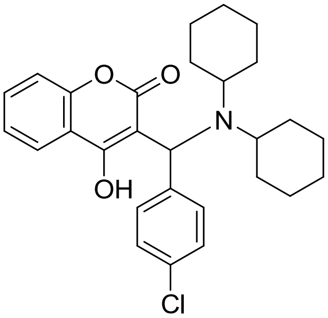

3-((4-chlorophenyl)(dicyclohexylamino)methyl)-4-hydroxy-2*H*-chromen-2-one

4b

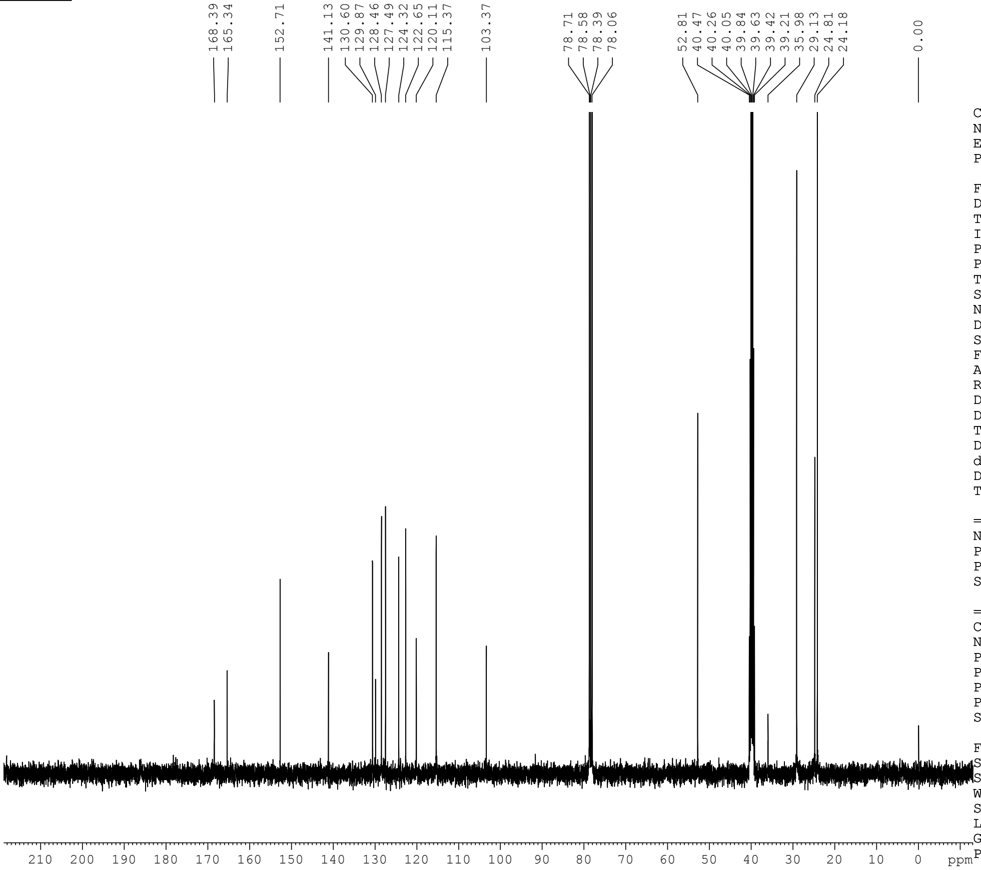

BRUKER
AVANCE II 400 NMR
Spectrometer
SAIF
Panjab University
Chandigarh

Current Data Parameters
NAME Jun13-2016
EXPNO 491
PROCNO 1

F2 - Acquisition Parameters
Date_ 20160614
Time 2.24
INSTRUM spect
PROBHD 5 mm PABBO BB-
PULPROG zgpg30
TD 65536
SOLVENT DMSO
NS 512
DS 4
SWH 29761.904 Hz
FIDRES 0.454131 Hz
AQ 1.1010548 sec
RG 2050
DW 16.800 usec
DE 6.00 usec
TE 297.9 K
D1 2.00000000 sec
d11 0.03000000 sec
DELTA 1.89999998 sec
TD0 1

======== CHANNEL f1 ========
NUC1 13C
P1 9.60 usec
PL1 -2.00 dB
SFO1 100.6228298 MHz

======== CHANNEL f2 ========
CPDPRG2 waltz16
NUC2 1H
PCPD2 80.00 usec
PL2 -3.00 dB
PL12 14.31 dB
PL13 18.00 dB
SFO2 400.1316005 MHz

F2 - Processing parameters
SI 32768
SF 100.6127961 MHz
WDW EM
SSB 0
LB 1.00 Hz
GB 0
PC 1.40

manishkumarmanu1986@gmail.com

4b

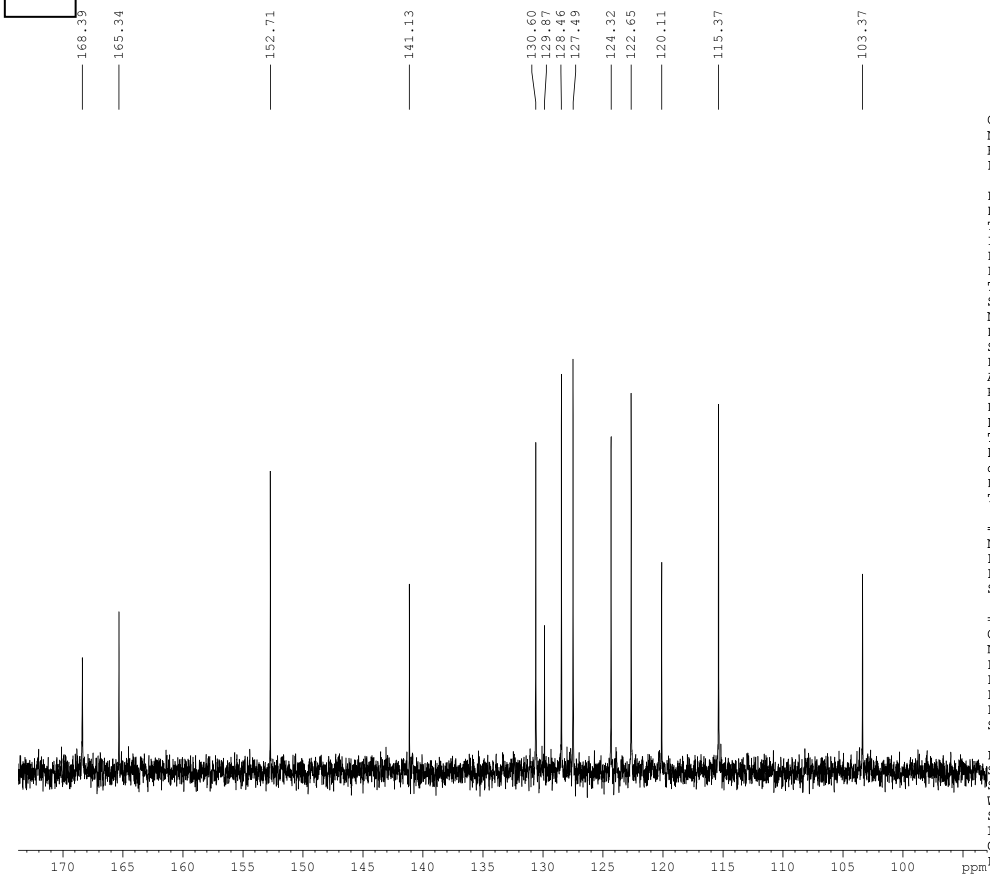

BRUKER
AVANCE II 400 NMR
Spectrometer
SAIF
Panjab University
Chandigarh

```
Current Data Parameters
NAME         Jun13-2016
EXPNO               491
PROCNO                1

F2 - Acquisition Parameters
Date_          20160614
Time               2.24
INSTRUM           spect
PROBHD   5 mm PABBO BB-
PULPROG         zgpg30
TD                65536
SOLVENT            DMSO
NS                  512
DS                    4
SWH          29761.904 Hz
FIDRES        0.454131 Hz
AQ           1.1010548 sec
RG                 2050
DW               16.800 usec
DE                 6.00 usec
TE                297.9 K
D1           2.00000000 sec
d11          0.03000000 sec
DELTA        1.89999998 sec
TD0                   1

======== CHANNEL f1 ========
NUC1                13C
P1                 9.60 usec
PL1               -2.00 dB
SFO1        100.6228298 MHz

======== CHANNEL f2 ========
CPDPRG2         waltz16
NUC2                 1H
PCPD2             80.00 usec
PL2               -3.00 dB
PL12              14.31 dB
PL13              18.00 dB
SFO2        400.1316005 MHz

F2 - Processing parameters
SI                32768
SF          100.6127961 MHz
WDW                  EM
SSB                   0
LB                 1.00 Hz
GB                    0
PC                 1.40
```

manishkumarmanu1986@gmail.com

Mass spectra of **4b**

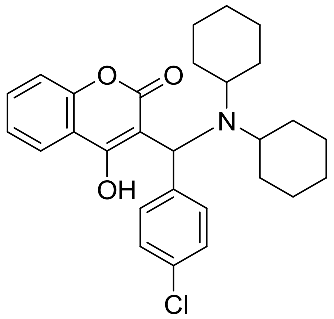

3-((4-chlorophenyl)(dicyclohexylamino)methyl)-4-hydroxy-2*H*-chromen-2-one

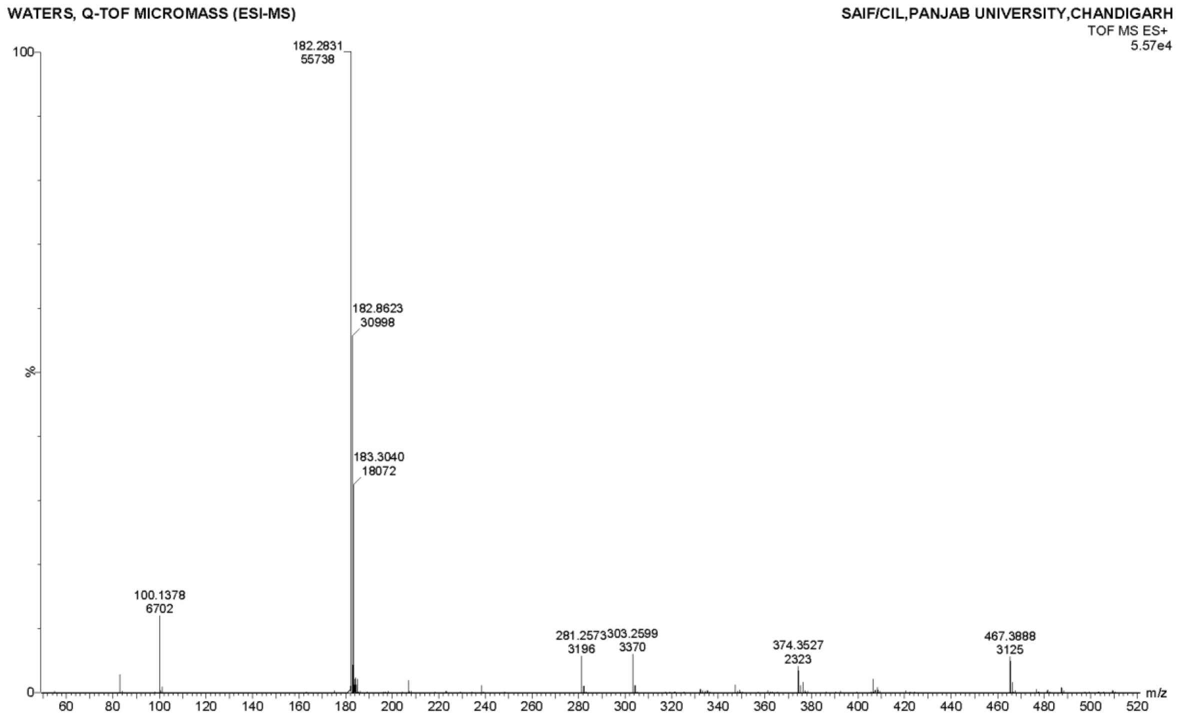

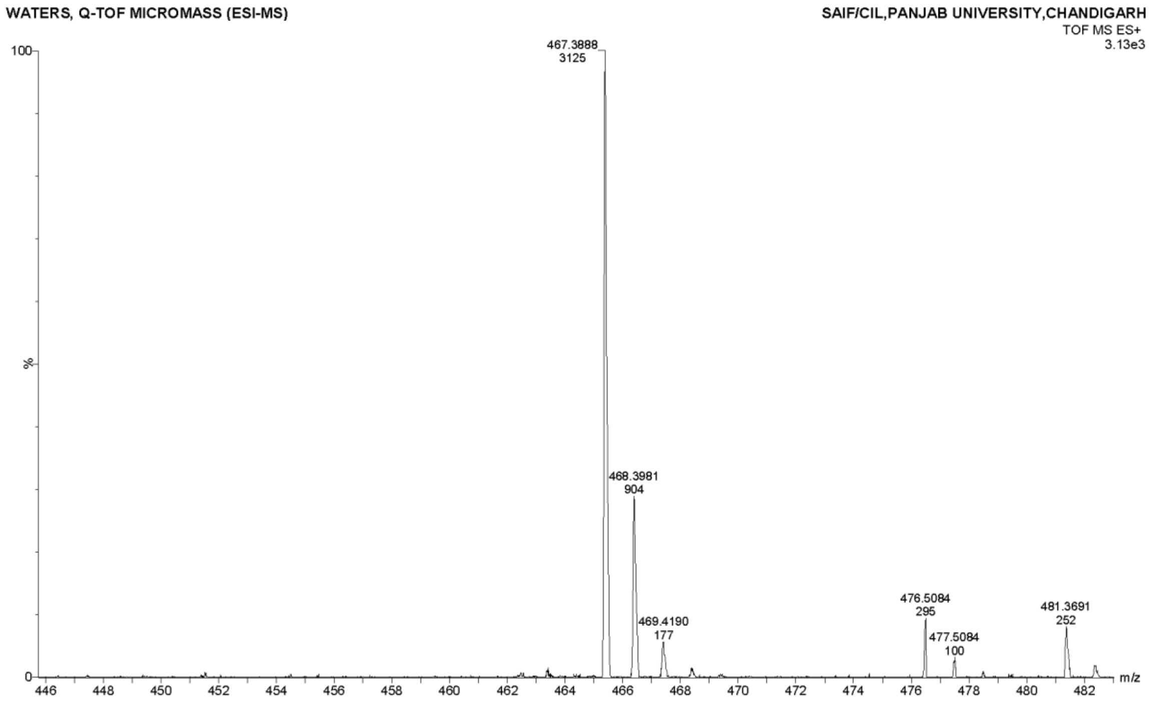

WATERS, Q-TOF MICROMASS (ESI-MS)
SAIF/CIL,PANJAB UNIVERSITY,CHANDIGARH
TOF MS ES+
3.13e3
467.3888
3125
468.3981
904
469.4190
177
476.5084
295
477.5084
100
481.3691
252
%
m/z

^{1}HNMRof **4f**

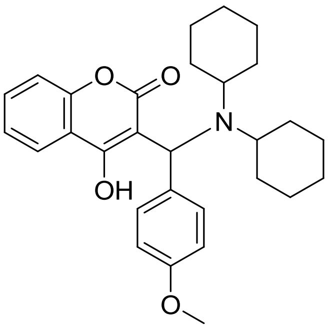

3-((Dicyclohexylamino)(4-methoxyphenyl)methyl)-4-hydroxy-2H-chromen-2-one

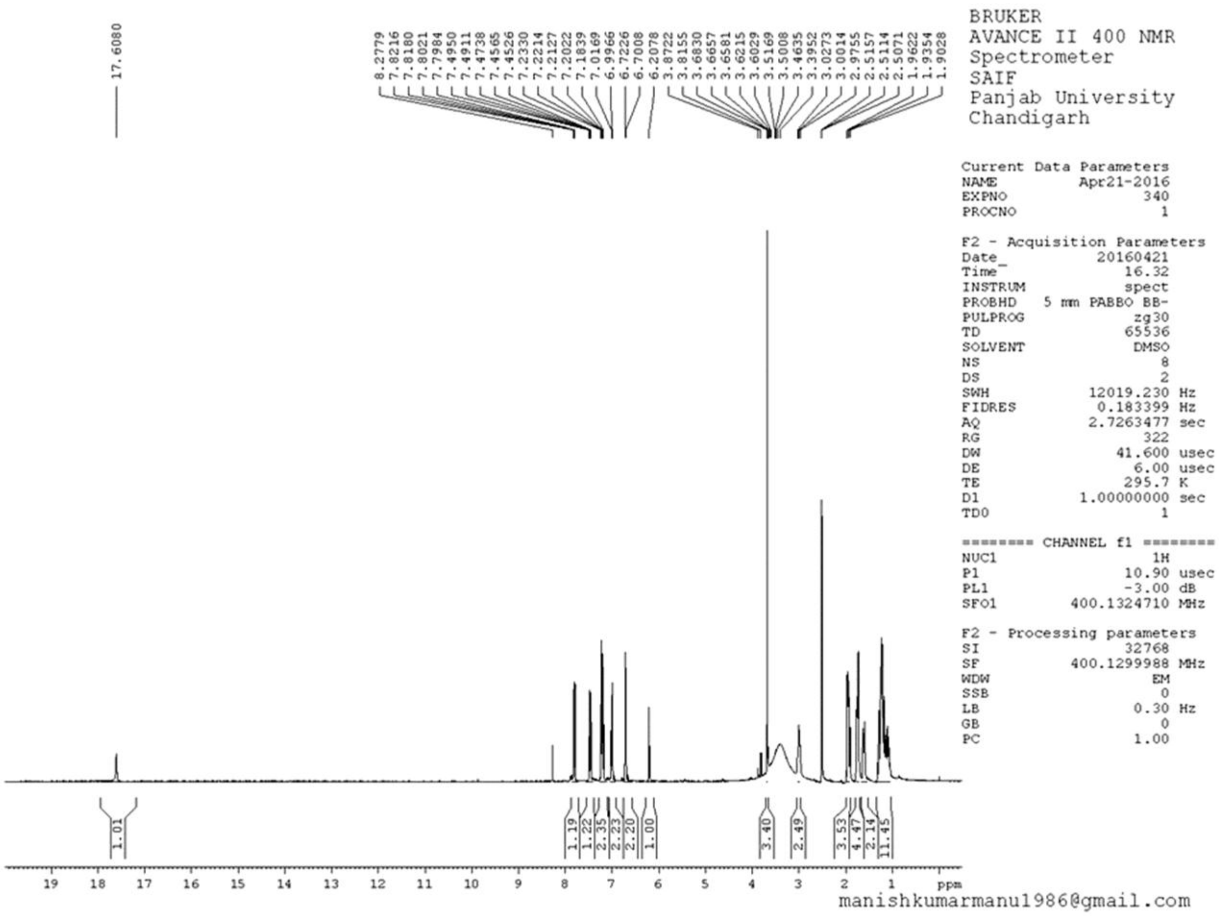

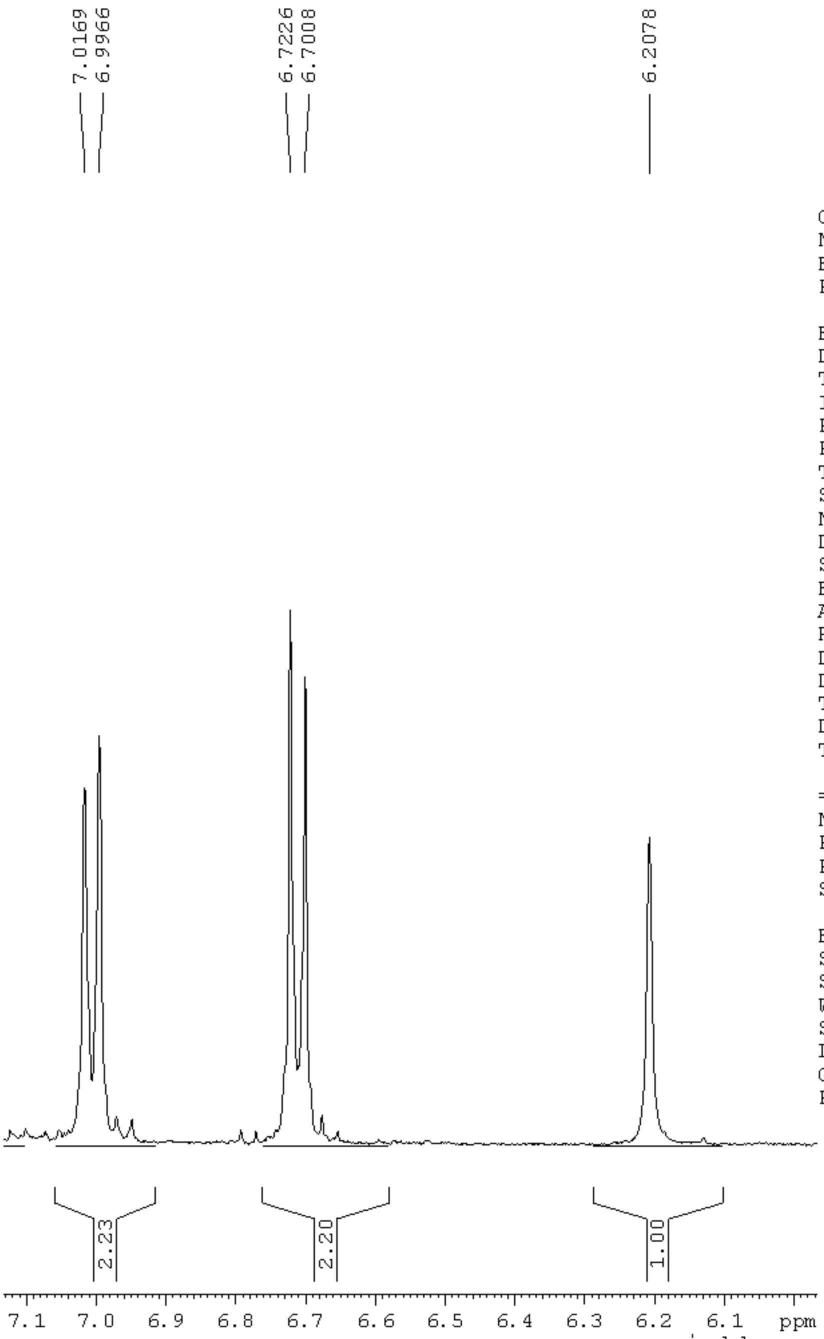

BRUKER
AVANCE II 400 NMR
Spectrometer
SAIF
Panjab University
Chandigarh

Current Data Parameters
NAME Apr21-2016
EXPNO 340
PROCNO 1

F2 - Acquisition Parameters
Date_ 20160421
Time 16.32
INSTRUM spect
PROBHD 5 mm PABBO BB-
PULPROG zg30
TD 65536
SOLVENT DMSO
NS 8
DS 2
SWH 12019.230 Hz
FIDRES 0.183399 Hz
AQ 2.7263477 sec
RG 322
DW 41.600 usec
DE 6.00 usec
TE 295.7 K
D1 1.00000000 sec
TD0 1

======== CHANNEL f1 ========
NUC1 1H
P1 10.90 usec
PL1 -3.00 dB
SFO1 400.1324710 MHz

F2 - Processing parameters
SI 32768
SF 400.1299988 MHz
WDW EM
SSB 0
LB 0.30 Hz
GB 0
PC 1.00

manishkumarmanu1986@gmail.com

^{13}CNMR of 4f

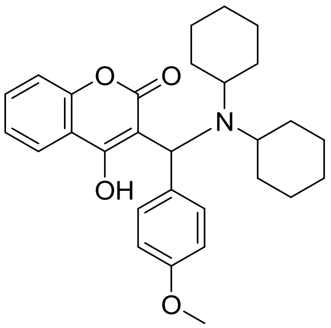

3-((Dicyclohexylamino)(4-methoxyphenyl)methyl)-4-hydroxy-2H-chromen-2-one

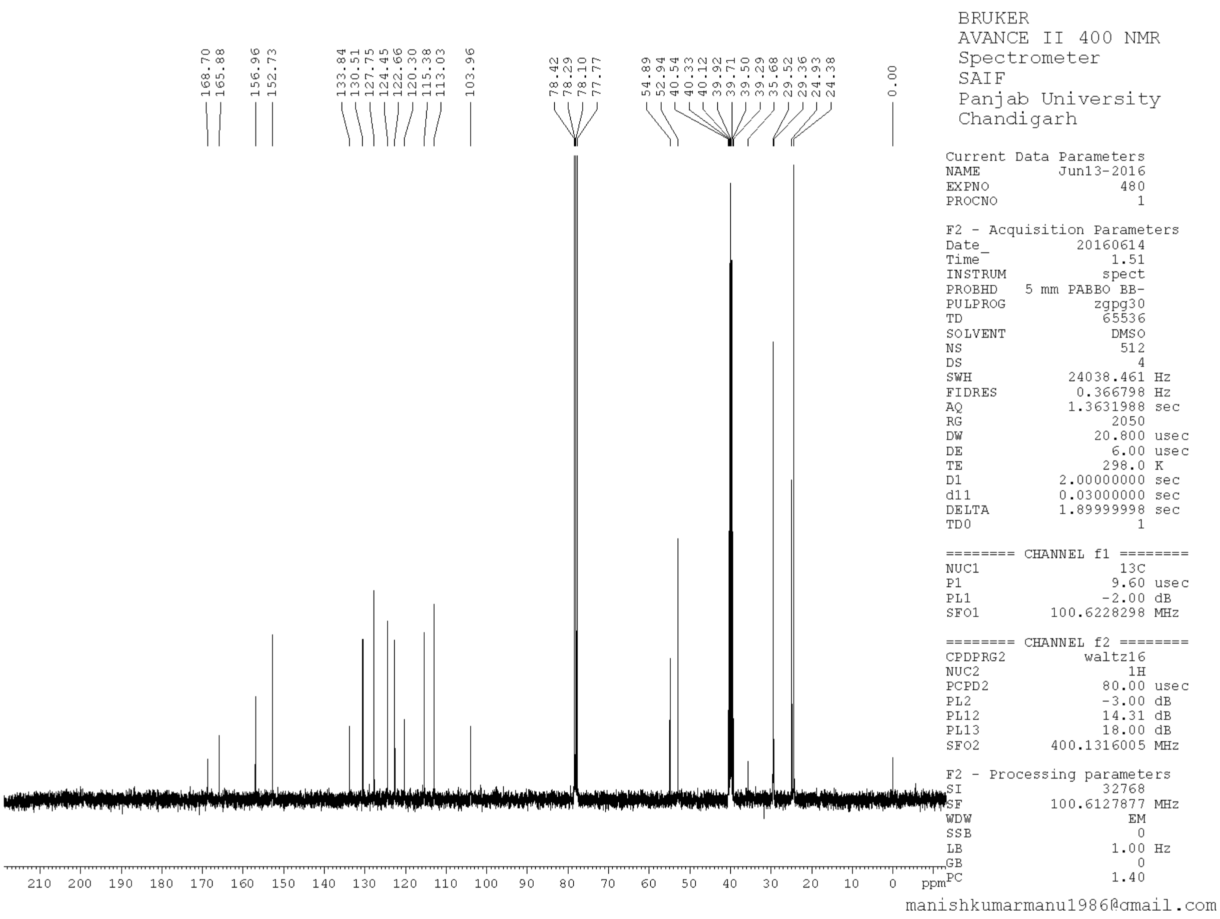

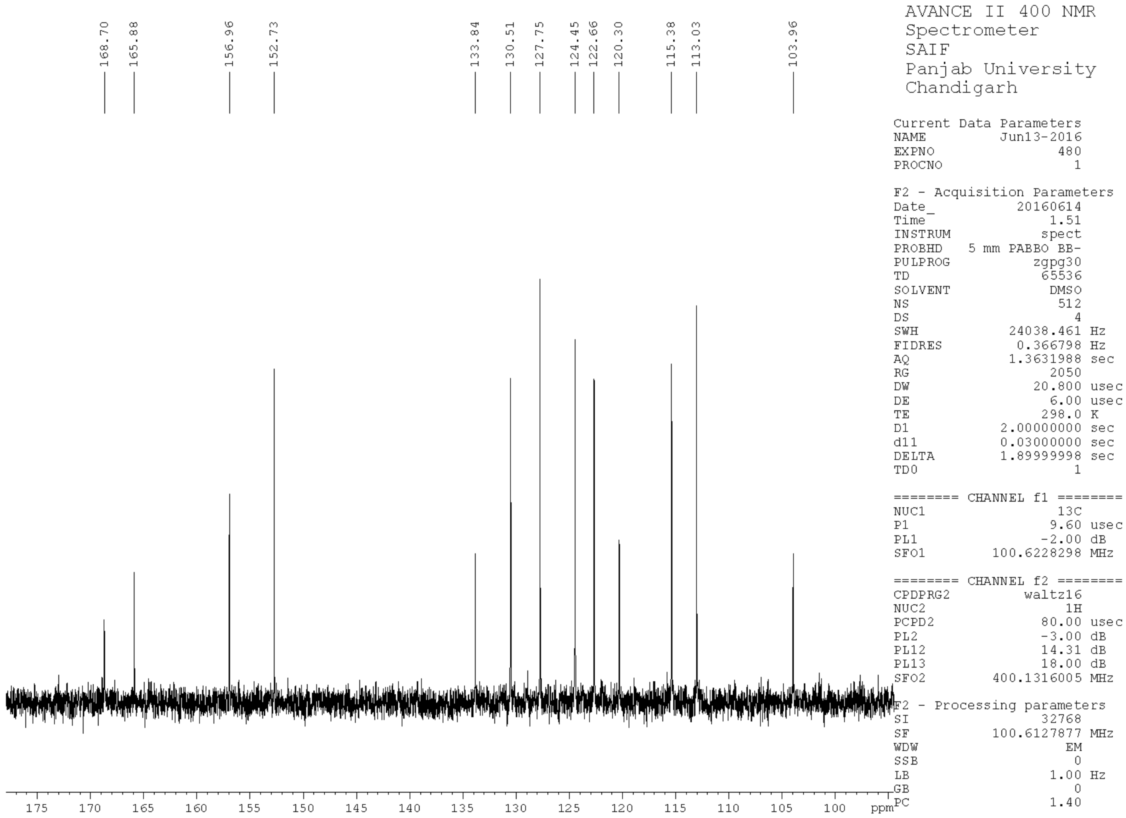

BRUKER
AVANCE II 400 NMR
Spectrometer
SAIF
Panjab University
Chandigarh

Current Data Parameters
NAME Jun13-2016
EXPNO 480
PROCNO 1

F2 - Acquisition Parameters
Date_ 20160614
Time 1.51
INSTRUM spect
PROBHD 5 mm PABBO BB-
PULPROG zgpg30
TD 65536
SOLVENT DMSO
NS 512
DS 4
SWH 24038.461 Hz
FIDRES 0.366798 Hz
AQ 1.3631988 sec
RG 2050
DW 20.800 usec
DE 6.00 usec
TE 298.0 K
D1 2.00000000 sec
d11 0.03000000 sec
DELTA 1.89999998 sec
TD0 1

======== CHANNEL f1 ========
NUC1 13C
P1 9.60 usec
PL1 -2.00 dB
SFO1 100.6228298 MHz

======== CHANNEL f2 ========
CPDPRG2 waltz16
NUC2 1H
PCPD2 80.00 usec
PL2 -3.00 dB
PL12 14.31 dB
PL13 18.00 dB
SFO2 400.1316005 MHz

F2 - Processing parameters
SI 32768
SF 100.6127877 MHz
WDW EM
SSB 0
LB 1.00 Hz
GB 0
PC 1.40

manishkumarmanu1986@gmail.com

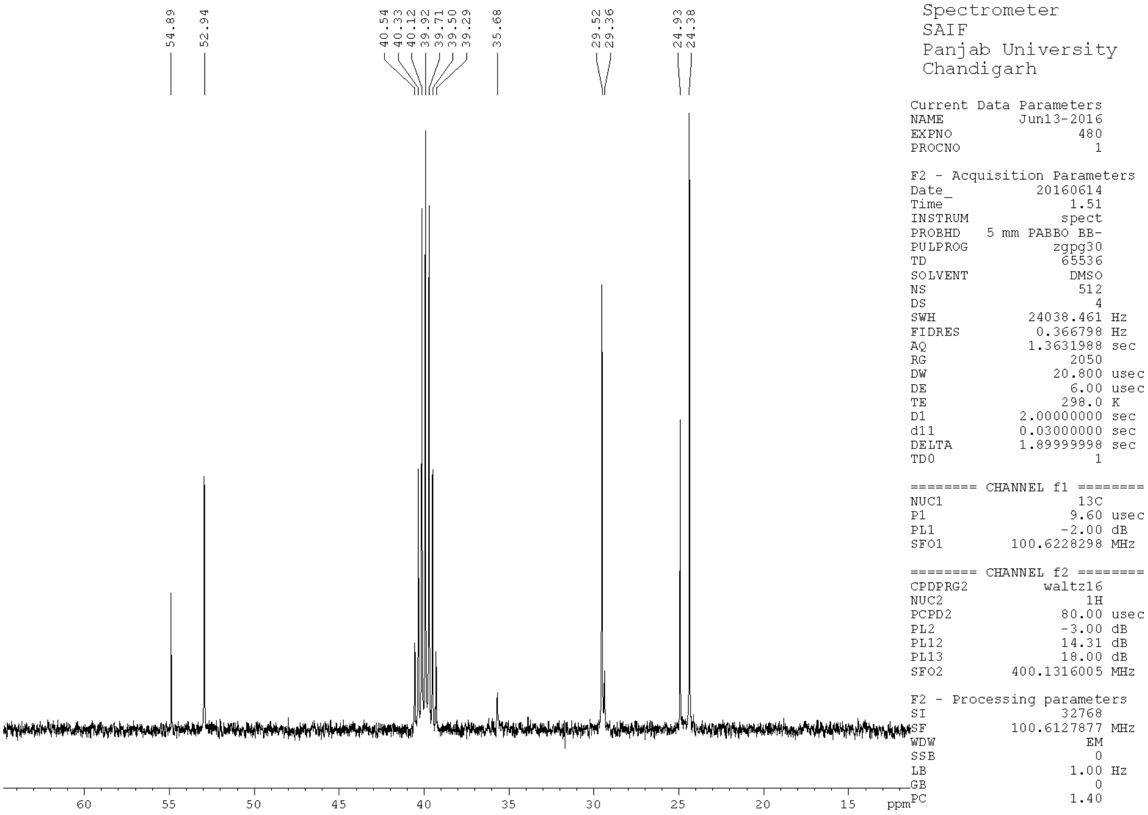

BRUKER
AVANCE II 400 NMR
Spectrometer
SAIF
Panjab University
Chandigarh

```
Current Data Parameters
NAME          Jun13-2016
EXPNO                480
PROCNO                 1

F2 - Acquisition Parameters
Date_           20160614
Time                1.51
INSTRUM            spect
PROBHD   5 mm PABBO BB-
PULPROG          zgpg30
TD                 65536
SOLVENT             DMSO
NS                   512
DS                     4
SWH            24038.461 Hz
FIDRES          0.366798 Hz
AQ             1.3631988 sec
RG                  2050
DW                20.800 usec
DE                  6.00 usec
TE                 298.0 K
D1            2.00000000 sec
d11           0.03000000 sec
DELTA         1.89999998 sec
TD0                    1

======== CHANNEL f1 ========
NUC1                 13C
P1                  9.60 usec
PL1                -2.00 dB
SFO1         100.6228298 MHz

======== CHANNEL f2 ========
CPDPRG2          waltz16
NUC2                  1H
PCPD2              80.00 usec
PL2                -3.00 dB
PL12               14.31 dB
PL13               18.00 dB
SFO2         400.1316005 MHz

F2 - Processing parameters
SI                 32768
SF           100.6127877 MHz
WDW                   EM
SSB                    0
LB                  1.00 Hz
GB                     0
PC                  1.40
```

manishkumarmanu1986@gmail.com

Mass spectra of **4f**

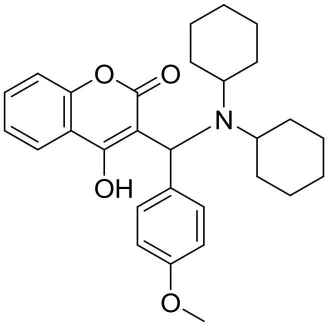

3-((Dicyclohexylamino)(4-methoxyphenyl)methyl)-4-hydroxy-2H-chromen-2-one

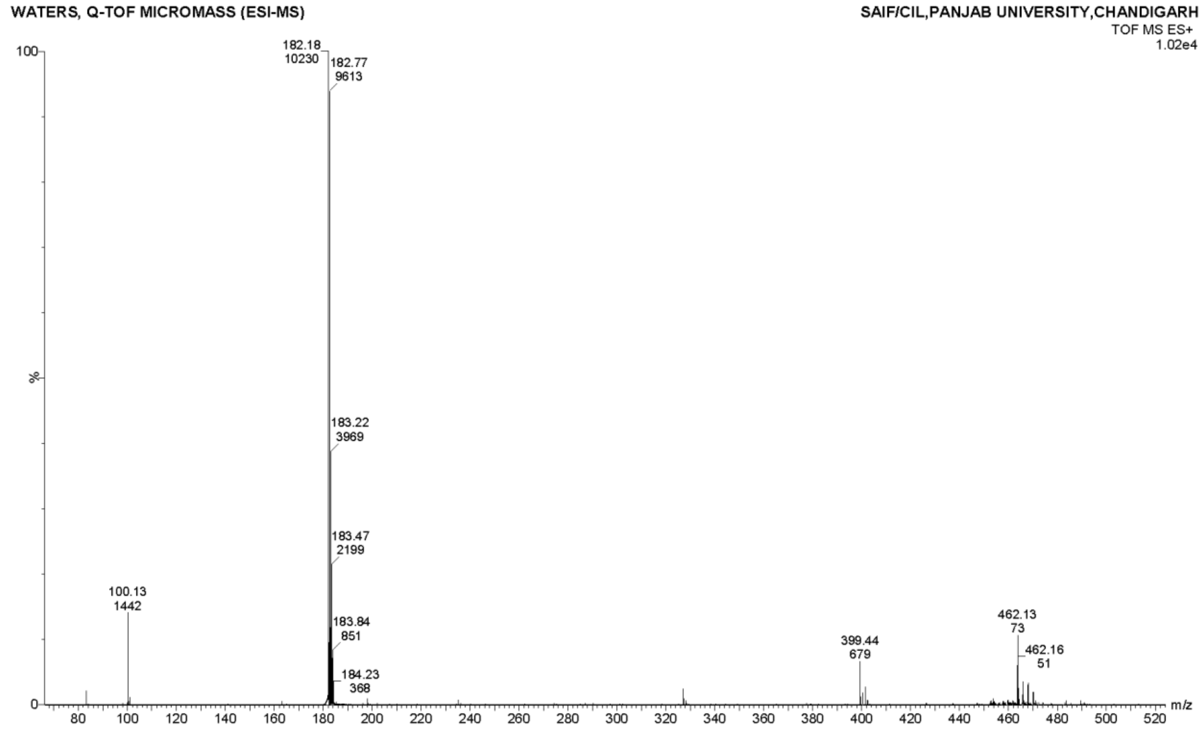

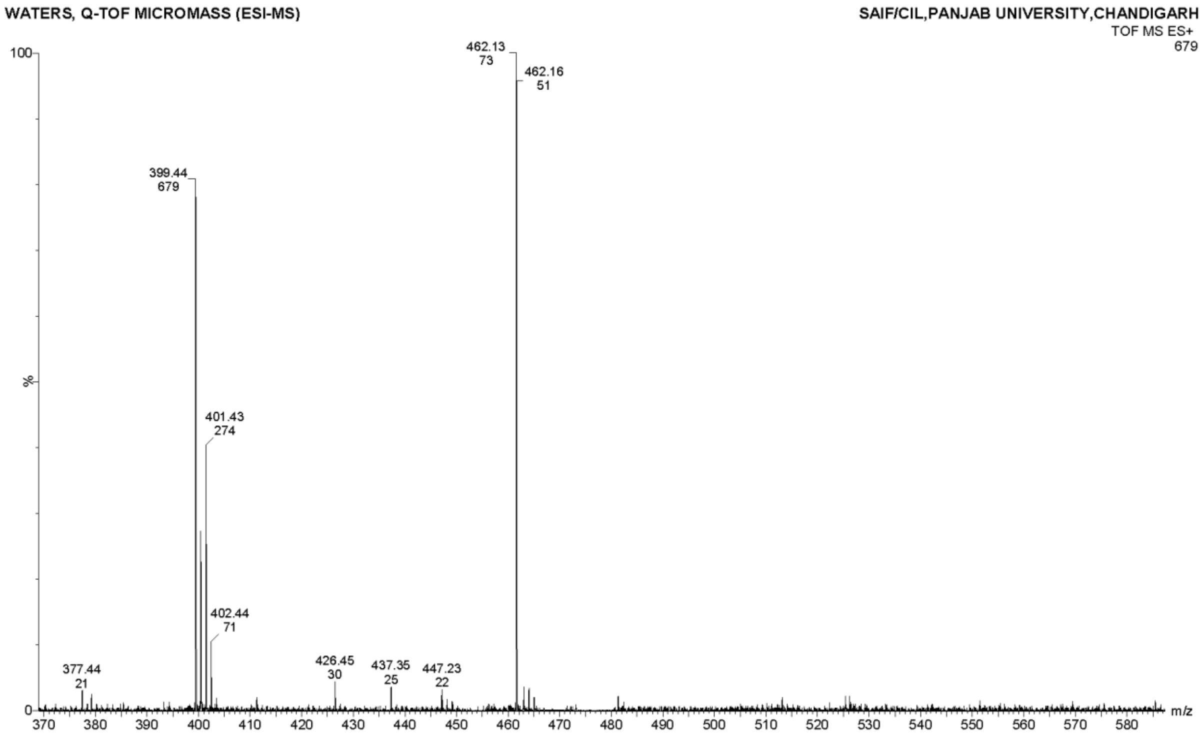
WATERS, Q-TOF MICROMASS (ESI-MS)
SAIF/CIL,PANJAB UNIVERSITY,CHANDIGARH
TOF MS ES+
679
100
%
0
462.13
73
462.16
51
399.44
679
401.43
274
402.44
71
377.44
21
426.45
30
437.35
25
447.23
22
370
380
390
400
410
420
430
440
450
460
470
480
490
500
510
520
530
540
550
560
570
580
m/z

^{1}HNMR **4n**

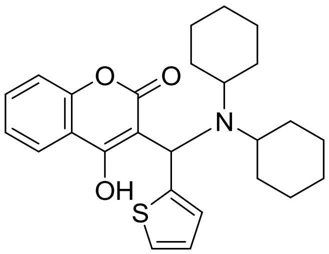

3-((Dicyclohexylamino)(thiophen-2-yl)methyl)-4-hydroxy-2H-chromen-2-one

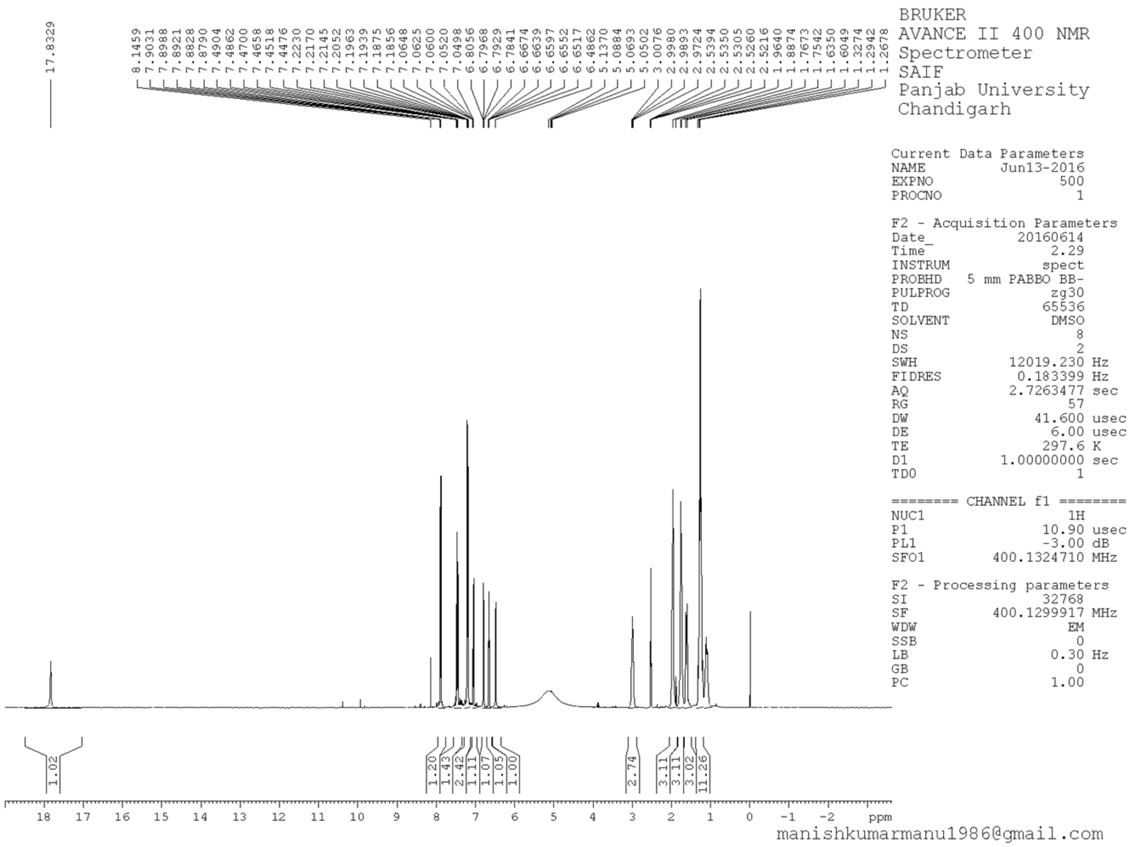

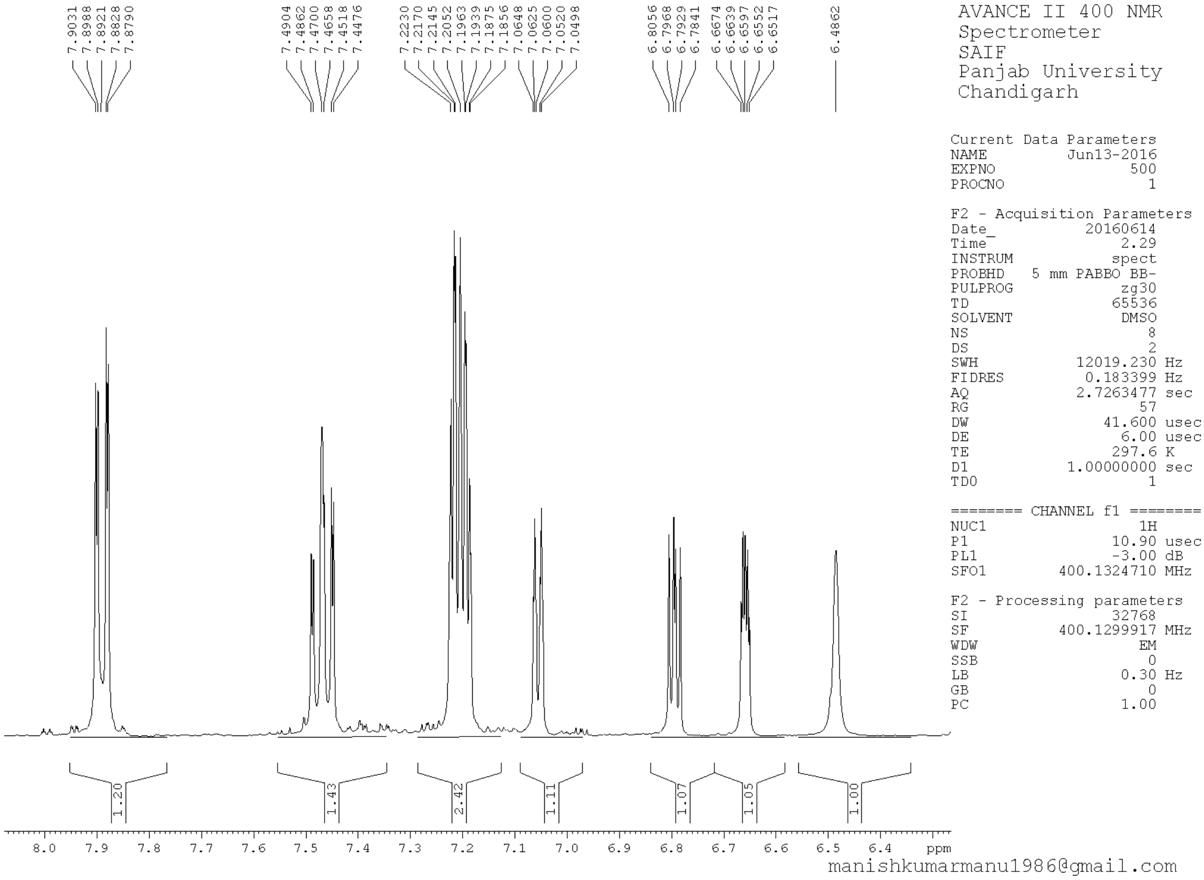

BRUKER
AVANCE II 400 NMR
Spectrometer
SAIF
Panjab University
Chandigarh

```
Current Data Parameters
NAME         Jun13-2016
EXPNO               500
PROCNO                1

F2 - Acquisition Parameters
Date_          20160614
Time               2.29
INSTRUM           spect
PROBHD   5 mm PABBO BB-
PULPROG            zg30
TD                65536
SOLVENT            DMSO
NS                    8
DS                    2
SWH           12019.230 Hz
FIDRES         0.183399 Hz
AQ            2.7263477 sec
RG                   57
DW               41.600 usec
DE                 6.00 usec
TE                297.6 K
D1           1.00000000 sec
TD0                   1

======== CHANNEL f1 ========
NUC1                 1H
P1                10.90 usec
PL1               -3.00 dB
SFO1        400.1324710 MHz

F2 - Processing parameters
SI                32768
SF          400.1299917 MHz
WDW                  EM
SSB                   0
LB                 0.30 Hz
GB                    0
PC                 1.00
```

manishkumarmanu1986@gmail.com

^{13}CNMR **4n**

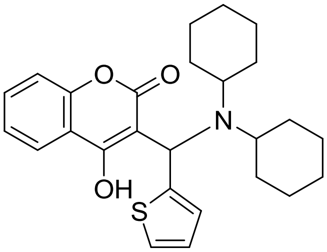

3-((Dicyclohexylamino)(thiophen-2-yl)methyl)-4-hydroxy-2H-chromen-2-one

4n

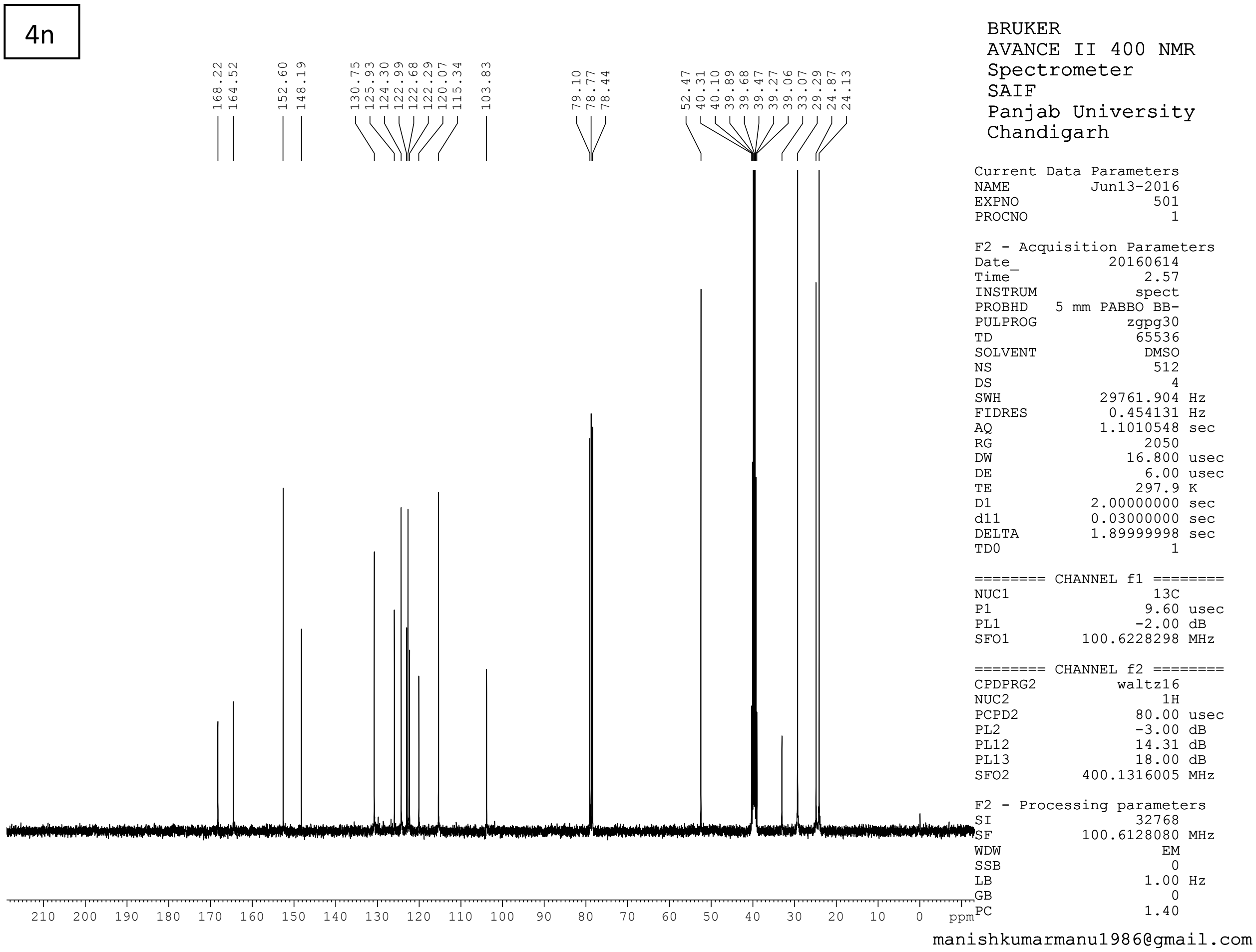

4n

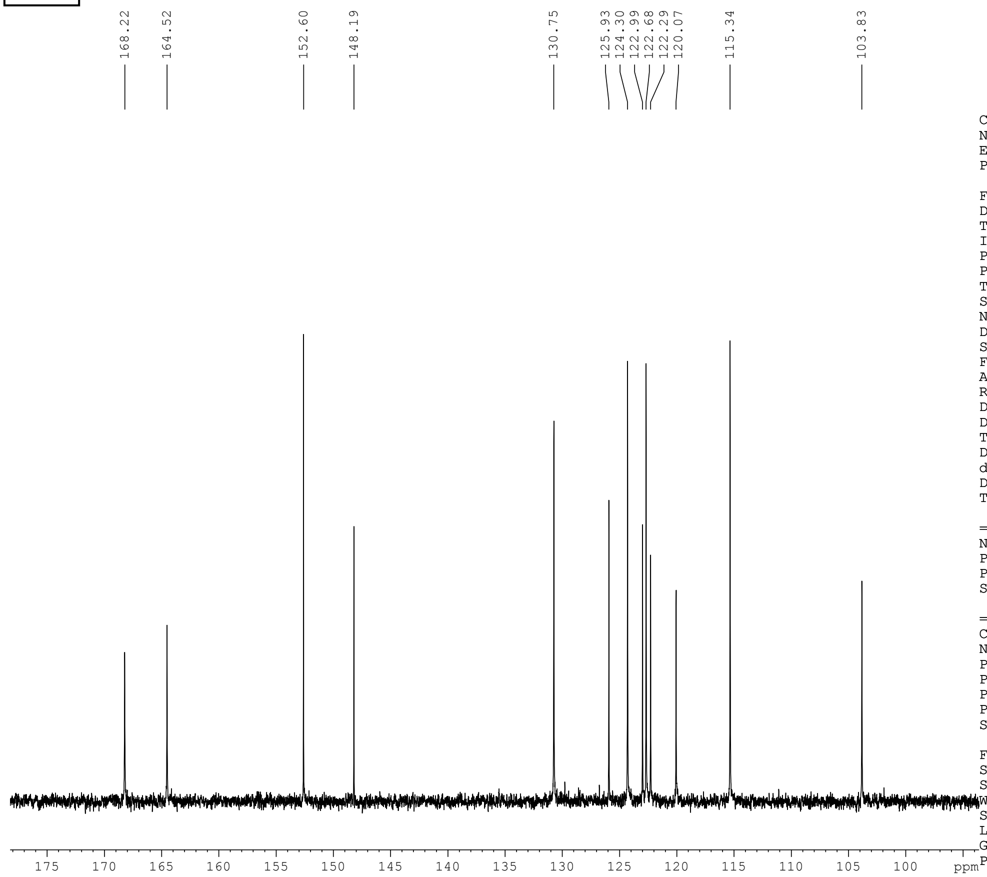

BRUKER
AVANCE II 400 NMR
Spectrometer
SAIF
Panjab University
Chandigarh

Current Data Parameters	
NAME	Jun13-2016
EXPNO	501
PROCNO	1
F2 - Acquisition Parameters	
Date_	20160614
Time	2.57
INSTRUM	spect
PROBHD	5 mm PABBO BB-
PULPROG	zgpg30
TD	65536
SOLVENT	DMSO
NS	512
DS	4
SWH	29761.904 Hz
FIDRES	0.454131 Hz
AQ	1.1010548 sec
RG	2050
DW	16.800 usec
DE	6.00 usec
TE	297.9 K
D1	2.00000000 sec
d11	0.03000000 sec
DELTA	1.89999998 sec
TD0	1
======== CHANNEL f1 ========	
NUC1	13C
P1	9.60 usec
PL1	-2.00 dB
SFO1	100.6228298 MHz
======== CHANNEL f2 ========	
CPDPRG2	waltz16
NUC2	1H
PCPD2	80.00 usec
PL2	-3.00 dB
PL12	14.31 dB
PL13	18.00 dB
SFO2	400.1316005 MHz
F2 - Processing parameters	
SI	32768
SF	100.6128080 MHz
WDW	EM
SSB	0
LB	1.00 Hz
GB	0
PC	1.40

manishkumarmanu1986@gmail.com

Mass spectra of **4n**

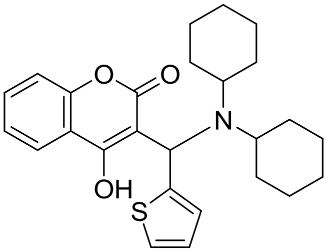

3-((Dicyclohexylamino)(thiophen-2-yl)methyl)-4-hydroxy-2H-chromen-2-one

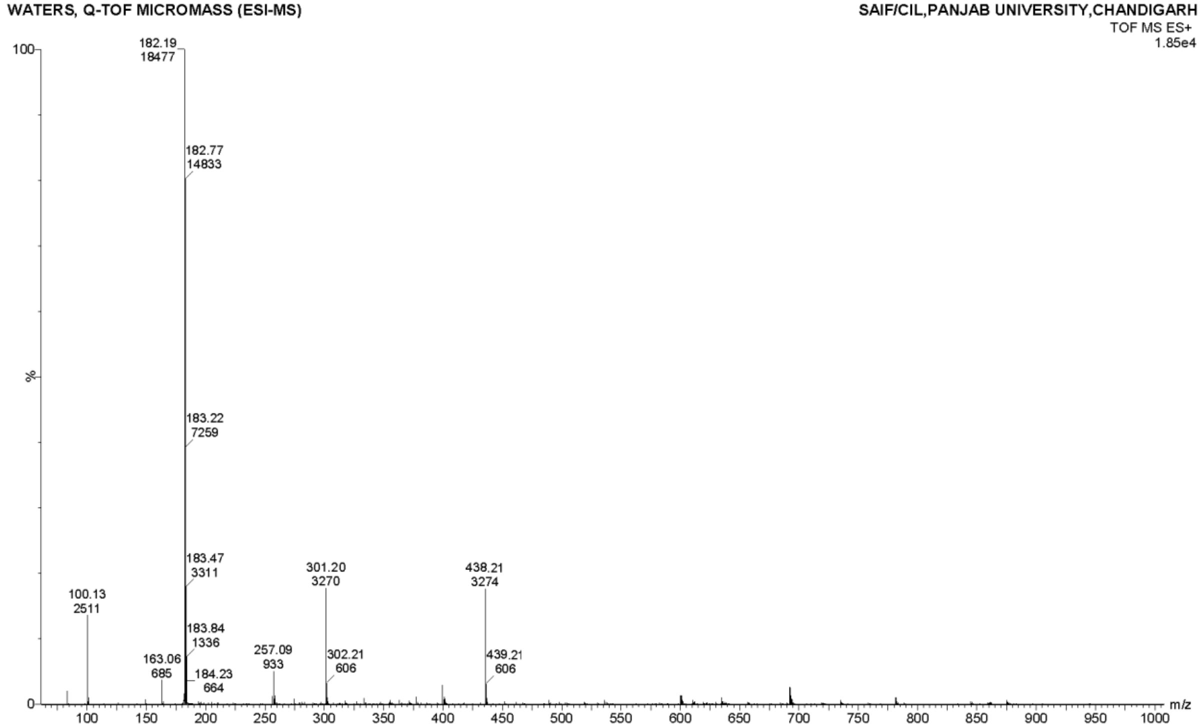

Supplement: Supplementary file 1 [file molecules-22-01172-s001.pdf]
